# Landscape of alternative splicing in *Capra_hircus*

**DOI:** 10.1038/s41598-018-33078-7

**Published:** 2018-10-11

**Authors:** Tieshan Xu, Feng Xu, Lihong Gu, Guang Rong, Mao Li, Fei Qiao, Liguang Shi, Dingfa Wang, Wanliang Xia, Wenjuan Xun, Ting Cao, Yiming Liu, Zhemin Lin, Hanlin Zhou

**Affiliations:** 1 Tropical Crop Genetic Resource Research Institute, Chinese Academy of Tropical Agricultural Sciences, Danzhou, Hainan P. R. China; 2 Institute of Animal Science & Veterinary, Hainan Academy of Agricultural Science, Haikou, P. R. China; 3 National Maize Improvement Center, China Agricultural University, Beijing, P. R. China

## Abstract

Alternative splicing (AS) is a fundamental regulatory process in all higher eukaryotes. However, AS landscapes for a number of animals, including goats, have not been explored to date. Here, we sequenced 60 samples representing 5 tissues from 4 developmental stages in triplicate using RNA-seq to elucidate the goat AS landscape. In total, 14,521 genes underwent AS (AS genes), accounting for 85.53% of intron-containing genes (16,697). Among these AS genes, 6,342 were differentially expressed in different tissues. Of the AS events identified, retained introns were most prevalent (37.04% of total AS events). Functional enrichment analysis of differential and specific AS genes indicated goat AS mainly involved in organ function and development. Particularly, AS genes identified in leg muscle were associated with the “regulation of skeletal muscle tissue development” GO term. Given genes were associated with this term, four of which (*NRG4*, *IP6K3*, *AMPD1*, and *DYSF*) might play crucial roles in skeletal muscle development. Further investigation indicated these five genes, harbored 13 ASs, spliced exclusively in leg muscle, likely played a role in goat leg muscle development. These results provide novel insights into goat AS landscapes and a valuable resource for investigation of goat transcriptome complexity and gene regulation.

## Introduction

The majority of eukaryotic genes are comprised of exons and introns. Their transcribed pre-mRNAs undergo RNA splicing where introns are excised and exons are joined together to form mature mRNA sequences. The exons and introns contained in pre-mRNAs can either be included or excluded from the mature mRNA through a process called alternative splicing (AS)^1^. AS was first discovered in the infectious adenovirus cycle^2,3^, it’s a fundamental regulatory process in the endogenous genes^4^ of all higher eukaryotes^5^.

AS is a widespread mechanism which increases transcriptome and proteome diversity and controls many biological processes in eukaryotes. Alternatively spliced mRNA isoforms encode different protein variants with altered amino acid sequences and therefore increase proteome diversity. It has been suggested that AS is involved in many biological processes, including several diseases in mammals^6^ and regulation of stress responses in plants^7^. In some cases, alternatively spliced mRNA can generate truncated proteins, which may interact with their partners to interfere with the formation of alternative homo- or hetero-dimers^8,9^. In addition, AS can regulate gene expression at the transcriptional and translational levels and can increase the complexity of microRNA-based gene regulation.

Previous studies found that ~35% of intron-containing genes in humans and ~5% of intron-containing genes in *Arabidopsis* underwent AS based on alignment of expressed sequence tag (EST) contigs to genomic DNA^10,11^. With the advent of tiling arrays and high-throughput sequencing, researchers found that 95% of intron-containing genes in humans^12^ and >60% of intron-containing plant genes undergo AS^13,14^. With recent advances in RNA isolation techniques^15^, sequencing techniques, and analysis tools (such as rMAT software for replicate RNA-Seq data)^16^, NGS-based RNA-seq datasets provide a rich resource for uncovering novel AS events and AS regulatory mechanism in a number of biological processes for different organisms.

Goats (*Capra_hircus*) serve as an important source of meat, milk, fiber, and pelts, and have also fulfilled agricultural, economic, cultural, and even religious roles throughout human civilization^17^. In addition, goats are now used as animal models for biomedical research, providing insights into the genetic basis of complex traits and transgenic production of peptides for medical purposes^18,19^, which greatly relies on our understanding of gene regulatory mechanisms. Therefore, investigation of goat gene regulatory mechanisms, including AS regulatory mechanisms, is especially important. The AS characteristics for several genes in goats have been investigated, including *Izumo1*^20^, *Lin28B*^21^, *GSK3β*^22^, and *NFIX*^23^. The goat draft genome (ftp://ftp.ncbi.nlm.nih.gov/genomes/Capra_hircus/)^24^ had been finished in 2013. The goat draft genome (CHIR 1.0) contains 2.52 G bases, 22,175 protein-coding genes and large number of ruminant-specific repeats, which cmprise 42.2% of the goat genome. The goat draft genome provides an excellent platform for genome-wide AS detection. However, investigation of the goat AS landscape on a genome-wide level has not been performed.

Here, we performed genome-wide detection and characterization of the AS landscape in goats using poly (A)+ RNA-seq data from five tissues across four developmental stages of Hainan Black goat in triplicate. Sequencing and analysis of these 60 samples allowed for detection and characterization of intron features, AS events, AS types, differential AS and functional enrichment analysis of genes undergoing AS at the genomic level for the first time in goats. Our results provide comprehensive insights into the goat AS landscape and a basis in further investigation of the functional roles of AS in goat gene expression.

## Results

### Overview of RNA-seq Data

**Figure 1:**
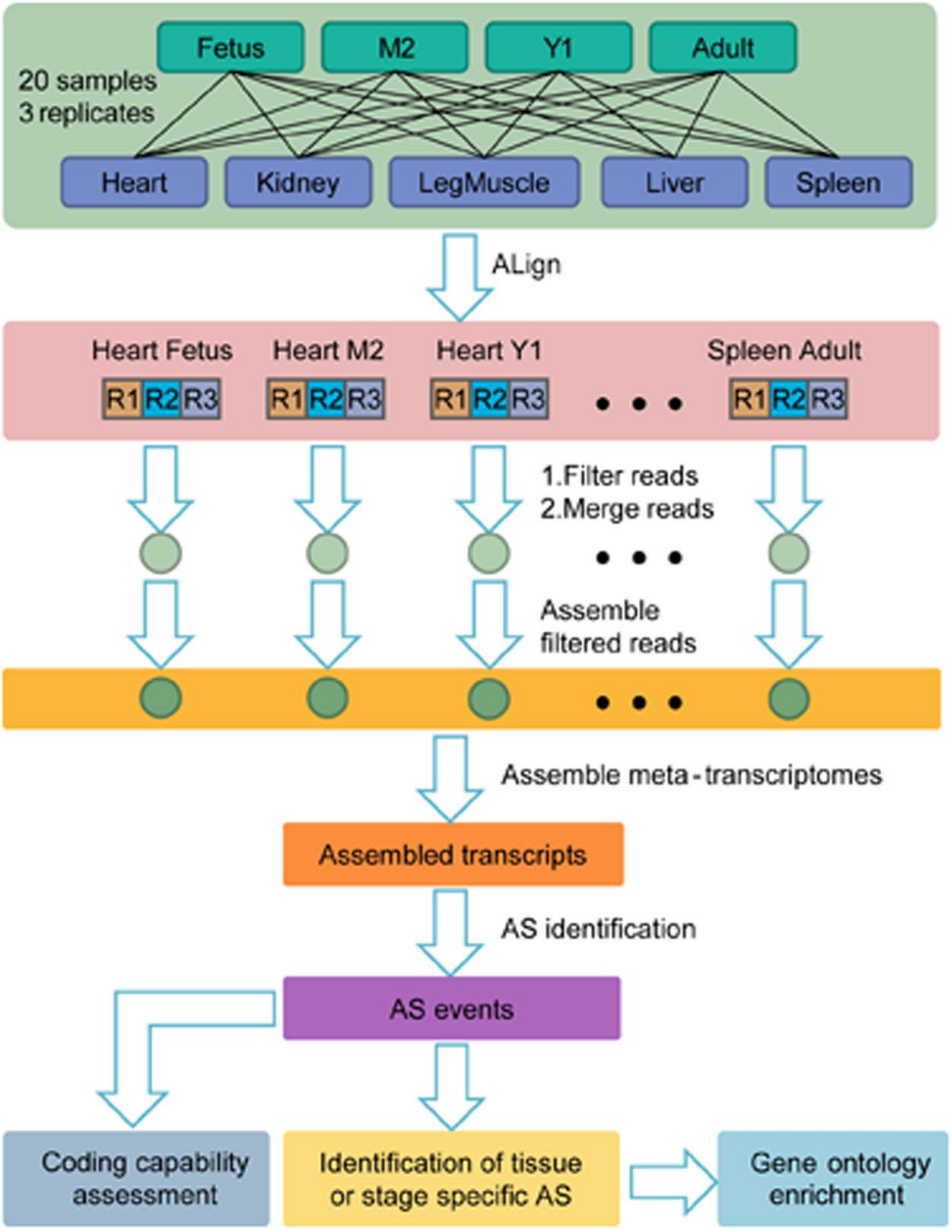
Pipeline for identifying goat AS events in this study. Fetus, M2, Y1, and Adult represent goats at embryonic stages beyond 135 d, two months of age, one year of age, and two years of age, respectively. Heart, Kidney, Leg muscle, Liver, and Spleen with three replicates (R1, R2, and R3) at each developmental stage were collected. After filtering and merging of raw reads, and assembling of filtered reads, assembled transcripts were obtained. After AS identification based on the assembled transcripts, coding capability assessment, and identification of tissues and developmental stage specific AS was performed. Gene ontology enrichment analysis for tissues and developmental stage specific AS were performed to identify the functional roles of AS genes.

To investigate the AS landscape in goats, we carried out high-throughput RNA-seq for 60 samples spanning five tissue types (heart, kidney, leg muscle, liver, and spleen) across four developmental stages (fetus, M2, Y1 and adult) from Hainan Black goat (Supplementary Table S1), utilizing a stringent pipeline to identify the AS landscape (Fig. 1). In total, 1.38 billion raw reads (344.16 Gb) were obtained, with an average of ~22.9 million raw reads (5.74 Gb) per sample. After filtering, a total of 1.35 billion high-quality reads (338.38 Gb) remained, representing an average of ~22.6 million (5.64 Gb) per sample (Supplementary Table S2). We then mapped the high-quality reads to the goat genome (CHIR1.0; ftp://ftp.ncbi.nlm.nih.gov/genomes/Capra_hircus/) using Tophat2^25^, which resulted in an alignment rate of 78.98%. Of the mapped reads, 97.14% mapped uniquely to one locus, while the remaining 2.86% mapped to multiple loci (Supplementary Table S3).

### Detection of splice junctions (SJs) and transcript assembly

**Figure 2:**
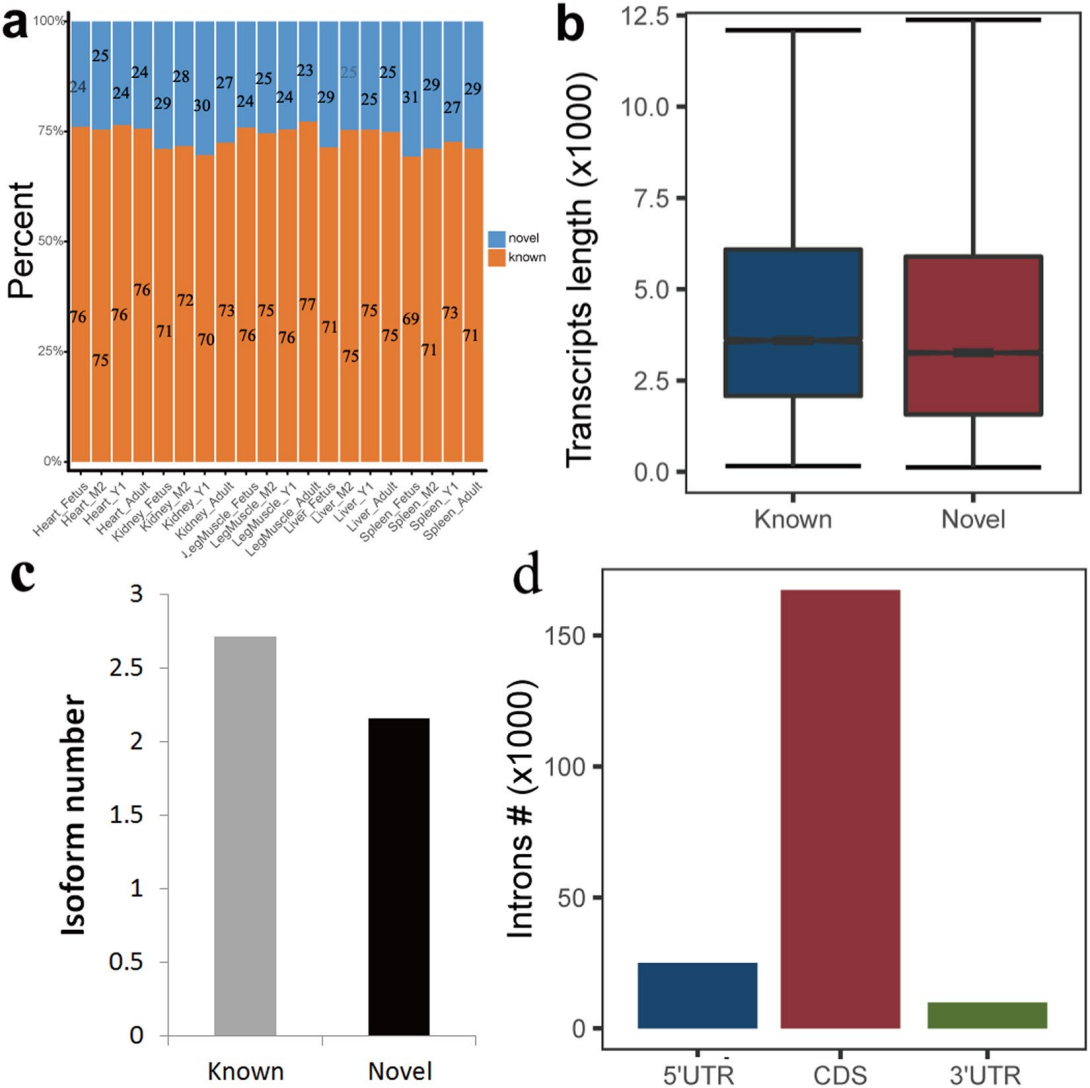
Characteristics of splice junctions (SJs) detected in this study. (**a**) The ratio of known (annotated in goat genome CHIR1.0) and novel (not annotated in goat genome CHIR1.0) SJs. (**b**) The comparison between known and novel goat transcript lengths. (**c**) Distribution of introns along annotated goat genes.

Transcript assembly is required for AS identification, and correct transcript assembly largely depends on accurate identification of SJs. Therefore, we performed SJ detection using TopHat2^26^. Initially obtained SJs were filtered according to two criteria described in detail in the methods. In total, we identified 680,911 SJs, including 479,361 novel SJs accounting for 70.4% of the total SJs. However, more than 70% of SJs were known SJs (annotated in goat genome) for each tissue (Fig. 2a). This is due to the majority of known SJs in a tissue always overlapped with that in another tissue, but the minority of novel SJs did not overlapped with that in another tissue. These results indicate the current SJs annotation in the goat genome (CHIR1.0) is largely incomplete.

To reduce the number of false positive assembled transcripts, we discarded transcripts that contained intronic reads >15% and displayed expression levels <10% of the major isoform from the same gene. Identified transcripts were then added to the goat gene annotation using Cuffcompare. In total, 55,035 genes (including 18,834 annotated genes and 36,201 novel genes) and 124,139 transcripts (including 83,489 annotated transcripts and 40,650 novel transcripts) were detected. In this study, we sequenced the cDNA fragment of transcribed RNAs with PolyA+, and tried to assemble them into full length transcripts. Because intergenic regions accounted for majority of goat genome and many long non-coding RNAs or other expression noise located in these regions, we detected 36,201 novel genes. However, almost all the current goat annotated genes were protein-coding genes in the paper of Dong *et al*.^24^, resulting in 22,175 protein-coding genes annotated. Thus, huge gap of gene counts existed between the current study and Dong *et al*.^24^. The average length and average isoform number distributions for known and novel transcripts are presented in Fig. 2b,c.

In total, we identified 16,997 intron-containing genes, of which 14,521 (85.53%) underwent AS (AS genes). This result is higher than estimated by the current goat genome, in which 78.33% of annotated intron-containing genes were AS genes (16,829 out of 21,484). These results indicated that the goat AS landscape is more complex than indicated in the annotated goat genome (CHIR1.0).

### Sequence characteristics of introns

In total, we detected 202,083 introns (Supplementary Table S4) for performance of downstream sequence analysis in order to investigate their sequence characteristics. The distribution of introns along goat annotated genes is presented in Fig. 2d. Overall, the majority of introns were spliced from coding sequence (CDS) genome regions.

**Figure 3:**
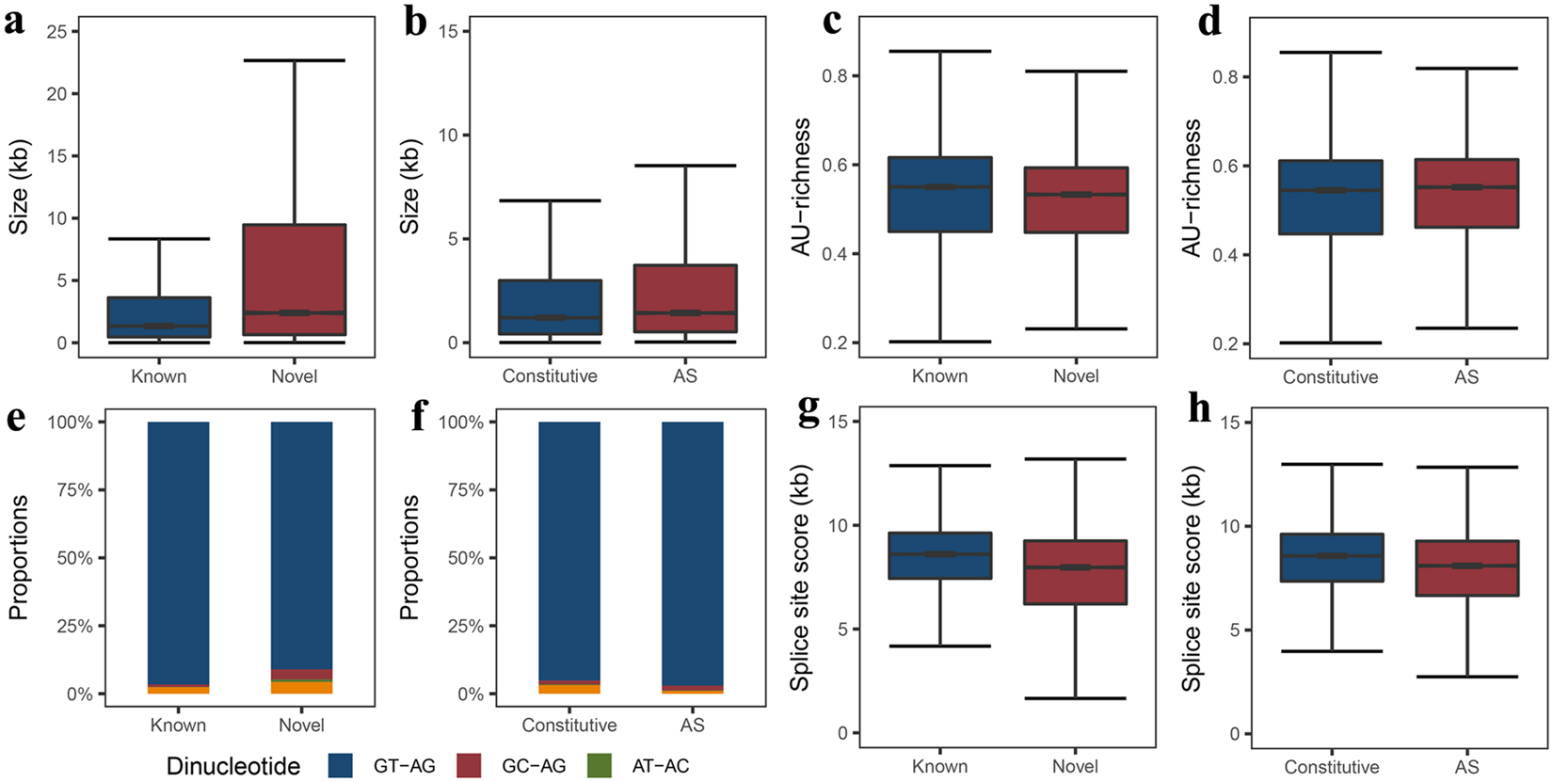
Characteristics of introns detected in this study. (**a** and **b**) Size comparison between known and novel introns, and between constitutive and alternatively spliced (AS) introns. (**c** and **d**) AU-richness comparison between known and novel introns, and between constitutive and AS introns. (**e** and **f**) Comparison of dinucleotide proportions between known and novel introns, and between constitutive and AS introns. (**g** and **h**) Comparison of splice site scores between known and novel introns, and between constitutive and AS introns.

Previous studies have demonstrated that several SJ characteristics affect the splice efficiency, including intron size, AU percentage, the dinucleotides at the intron borders, and the sequence of the 5′ and 3′ splice sites^27,28^. We therefore investigated these sequence characteristics in the identified goat introns, the results of which are presented in Fig. 3a–h and Supplementary Table S4.

The average length of the predicted introns was 6,103.75 nt (median = 1,472 nt; Fig. 3a,b and Supplementary Table S4). The average length of introns identified in this study is much longer than in the goat genome annotation (average = 3,955.00 nt, median = 1,347.00 nt respectively) (wilcoxon rank sum test, *P*-value = 1.2 e-111)^24^ and the results of Hawkins *et al*.^29^. In addition, the intron sizes produced by novel SJs (mean = 10,720.00 nt, median = 2,399.00 nt) is longer than produced by known SJs (wilcoxon rank sum test, *P*-value = 0), which may indicate that introns in genes undergoing AS tend to be larger than introns in genes that do not undergo AS (Supplementary Table S4). Furthermore, we identified 4,515 (~2% of total predicted introns) enormous introns in our study with a length >50 kb (Supplementary Table S4). In addition, we also calculated the intron sizes of other five species according to the GFF annotation files and genome sequences (Supplementary Table S5). The results indicated that the average intron size of goat identified in this study was much shorter than that of human and slightly shorter than that of mouse, but much longer than those of chicken, lizard, and frog.

The AU-richness of introns contributes to intron recognition and splicing efficiency^27^. We therefore examined the AU-richness of identified introns (Fig. 3c,d and Supplementary Table S4). The AU-richness of introns in genes undergoing AS was slightly higher (53.40%) than introns in genes that do not undergo AS (51.96%) (wilcoxon rank sum test, *P*-value = 4.3 e-35). The AU-richness of known introns (annotated in goat genome CHIR1.0) was 52.46%, which was slightly higher than that of novel introns (51.22%) (wilcoxon rank sum test, *P*-value = 3.72 e-153). The AU-richness of introns in this study was consistent with that of human, mouse and chicken, but much lower than that of lizard and frog (Supplementary Table S5). The AU-richness of introns in this study was also much lower than the results of Marquez *et al*.^13^ and Yu *et al*.^14^ in *Arabidopsis*, who both reported intron AU-richness higher than 60%^13,14^.

We then examined the dinucleotides at intron borders (Fig. 3e,f). The results indicated GT-AG sequences made up the majority of dinucleotides at the intron borders, which accounted for >95% of total dinucleotides. The percentages of GT-AG for known introns were slightly higher than novel introns. Our results were consistent with the results of Marquez *et al*. (2012) and Yu *et al*.^14^ in *Arabidopsis*^13,14^.

Finally, we compared the splice site scores (how similar the splice sites fits the consensus sequence) for constitutive introns, AS introns, known introns, and novel introns to explore differences in splicing power (Fig. 3g,h and Supplementary Table S4). The average splice site score for constitutive introns (mean = 8.09, median = 8.53) was slightly higher than that of AS introns (mean = 7.65, median = 8.10) (wilcoxon rank sum test, *P*-value < 1e-255). Similarly, the average splice site score for known introns (mean = 8.30, median = 8.61) was higher than that of novel introns (mean = 6.86, median = 7.97) (wilcoxon rank sum test, *P*-value < 1e-255), which may due to easier identification of introns with strong splicing power.

### AS types and distribution

**Figure 4:**
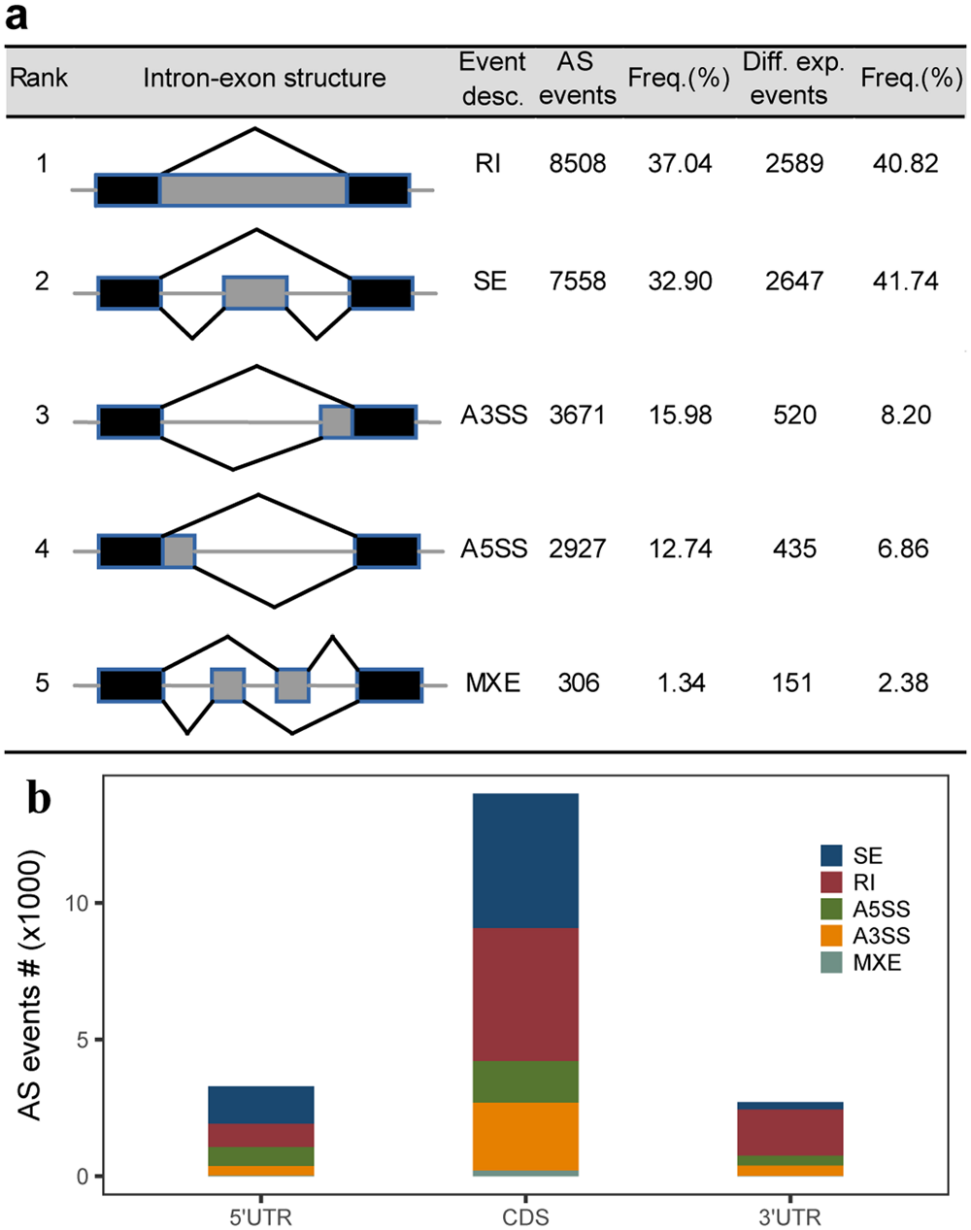
Types and distribution of five major AS events detected in this study. (**a**) Identified AS events and their frequencies, and differentially existed AS events and their frequencies. SE represents skipped exon AS events, RI represents retained intron AS events, A5SS represents alternative 5′ splice site AS events, A3SS represents alternative 3′ splice site AS events, and MXEs represents mutually exclusive exon AS events. (**b**) The distribution of AS events and types in different regions of goat annotated genes.

To account for the effect of biological variability, all the samples of a specific stage were collected from three different goats. The types and distribution of AS events in our dataset were determined using rMATS software^16^, which supports AS detection using RNA-seq data with biological replicates. In this study, we considered five major AS types described in^12^, including skipped exons (SE), retained introns (RI), alternative 5′ splice sites (A5SS), alternative 3′ splice sites (A3SS), and mutually exclusive exons (MXEs). We identified a total of 22,970 AS events across 8,460 genes belonging to one of the five AS types (Supplementary Table S6). RI was the most common AS event (8,508), accounting for 37.04% of the total AS events (Fig. 4a and Supplementary Table S6). SE was the second most prevalent AS event (32.90%). Our results also indicate that A3SS and A5SS account for a considerable amount of AS events (15.98% and 12.74% respectively), while MXE is a rare event (1.34%) (Fig. 4a). We also calculated the frequency of the five main AS types in humans, frogs, and lizards using the online human RNA-seq data from human (GSE45237), frog and lizard (GSE41338), and compared them to the results obtained in this study (Table 1). The most significant difference in frequency of AS type between goats and other species was that RI was the most commonly observed AS event in goats, but only accounted for a small portion in other species. In addition, SE was the most common AS type observed in humans, frogs, and lizards, while SE was the second most common AS type in goats. The results above indicate that the goat AS landscape is different from other species^30,31^, further investigation is required to explore the mechanism underlying these differences.

**Table 1:** Comparison of AS frequencies across four species for the five main AS types.

AS Types	SE/%	A3SS/%	A5SS/%	RI/%	MXE/%
Species					
Human	47.04	22.48	14.87	14.59	1.02
Goat	32.90	15.98	12.74	37.04	1.33
Frog	51.82	17.28	12.48	14.50	3.92
Lizard	68.61	12.48	8.35	6.11	4.45

Subsequently, we assessed the distribution of AS types along annotated genes. We presumed the longest isoform of a gene was the representative isoform, divided the gene into three different regions (5′ UTR, CDS, and 3′ UTR), and counted the number of AS events by type in each region. Results indicated that the majority of AS events (14,001) fell in CDS, among which SE and RI accounted for 35.09% and 34.75%, respectively, with the other three AS types accounting for ~30%. A considerable number of AS events occurred in 5′ UTRs and 3′ UTRs (3,292 and 2,715, respectively). The RI events occurred at a higher frequency in 3′ UTRs (1,692; 62.32%) than 5′ UTRs (849; 25.79%), while SE events were more frequent in 5′ UTRs than 3′ UTRs (41.56% VS 9.65%, respectively) (Fig. 4b).

### Differential AS events (DASE)

**Figure 5:**
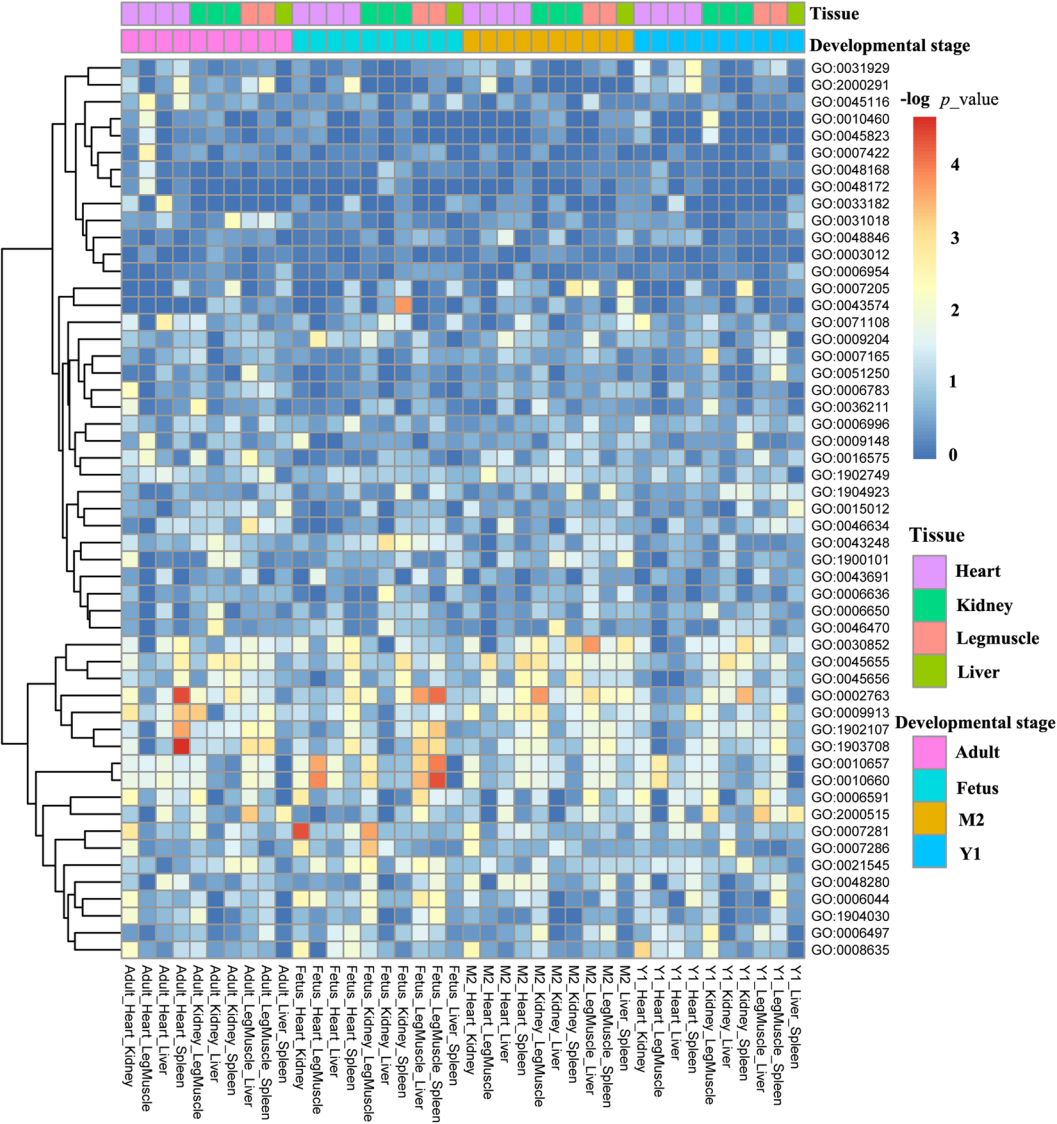
Differential alternative splicing events (DASE) across tissues at the same development stage.

**Figure 6:**
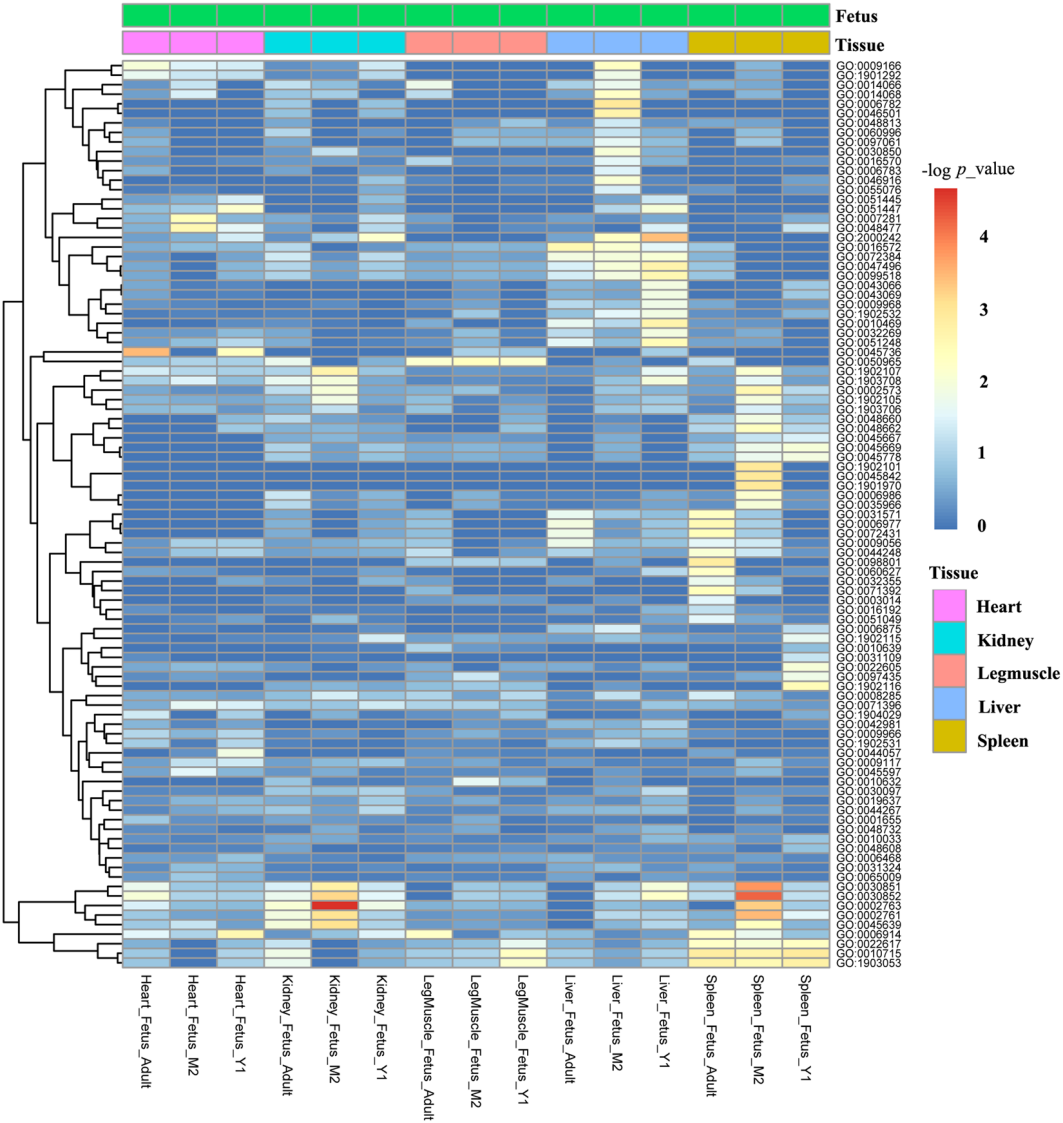
The DASE across developmental stages for the same tissue.

We identified DASE across tissues at the same development stage, and across developmental stages for the same tissue using a likelihood-ratio test in the rMATS package^16^ followed by GO term functional enrichment analysis. DASE across tissues at the same developmental stage were found to be involved in the functional maintenance of organs (Fig. 5 and Supplementary Table S7). For example, DASE between heart and leg muscle at the adult timepoint were mainly enriched in positive regulation of heart rate or heart contraction. DASE between leg muscle and liver, and between leg muscle and spleen at the fetal timepoint were mainly enriched in regulation of muscle cell apoptosis or regulation of leukocyte differentiation. While DASE across tissues were related to organ maintenance, the DASE across developmental stages of the same tissue were related to different physiological stages (Fig. 6 and Supplementary Table S8). Looking at the DASE across developmental stages in spleen as an example, 11 of the 15 enriched GO terms between fetus and M2 are associated with the regulation of cell growth, differentiation, and proliferation, while DASE between fetus and Y1 were involved in the regulation of organelle assembly and organization.

### Tissue- and developmental stage- specific AS

It has been shown that most AS events display strong specificity to a particular tissue or developmental stage^5,32,33^. In this study, we explored the tissue- and developmental stage- specific AS events to assess the extent of regulation specific to tissues and developmental stages.

**Figure 7:**
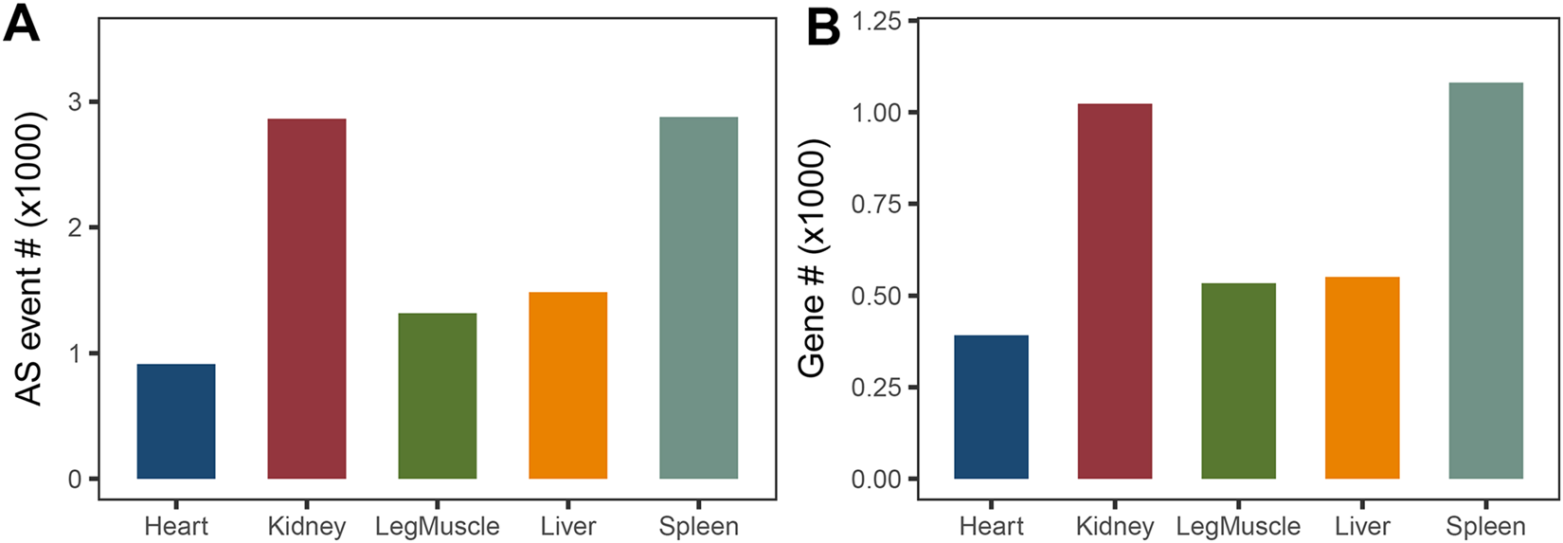
Tissue- specific AS events and AS genes. (**A**) Tissue- specific AS events in different tissues. (**B**) Tissue- specific AS genes in different tissues.

To investigate tissue-specific AS events, we first combined the samples from the same tissue at various developmental stages. We then compared the specificity using the Tau (τ) method^34^. Of the 43,396 AS events, 9,463 spanning 3,580 genes were located exclusively in one tissue (Fig. 7A,B and Supplementary Data S1). The majority of tissue-specific AS events located in spleen and kidney, with 2,879 and 2,864 AS events, respectively (representing 1,081 and 1,023 AS genes respectively). Substantial tissue-specific AS events were observed in liver and leg muscle as well, corresponding to 1,487 and 1,319 AS events in 550 and 534 genes, respectively. The fewest number of tissue specific AS events were observed in the heart.

We further investigated developmental stage-specific AS events for each tissue to explore the regulatory potential of AS in development (Supplementary Data S2). The results indicated there are very few AS events specifically located in a development specific manner, with only 35, 177, 87, 249, and 130 AS events specifically located in various stages of heart, kidney, leg muscle, liver, and spleen development, respectively. This suggests that the majority of tissue-specific AS events are specifically existed across multiple developmental stages, and that the majority of AS events are tissue-specific as opposed to developmental stage-specific. In addition, more AS events were found to be specifically located in fetus tissues, with the exception of fetal heart, indicating fetal tissues appear different AS profiles compared to postnatal stages.

### AS genes may be involved in the function and development of organs

**Figure 8:**
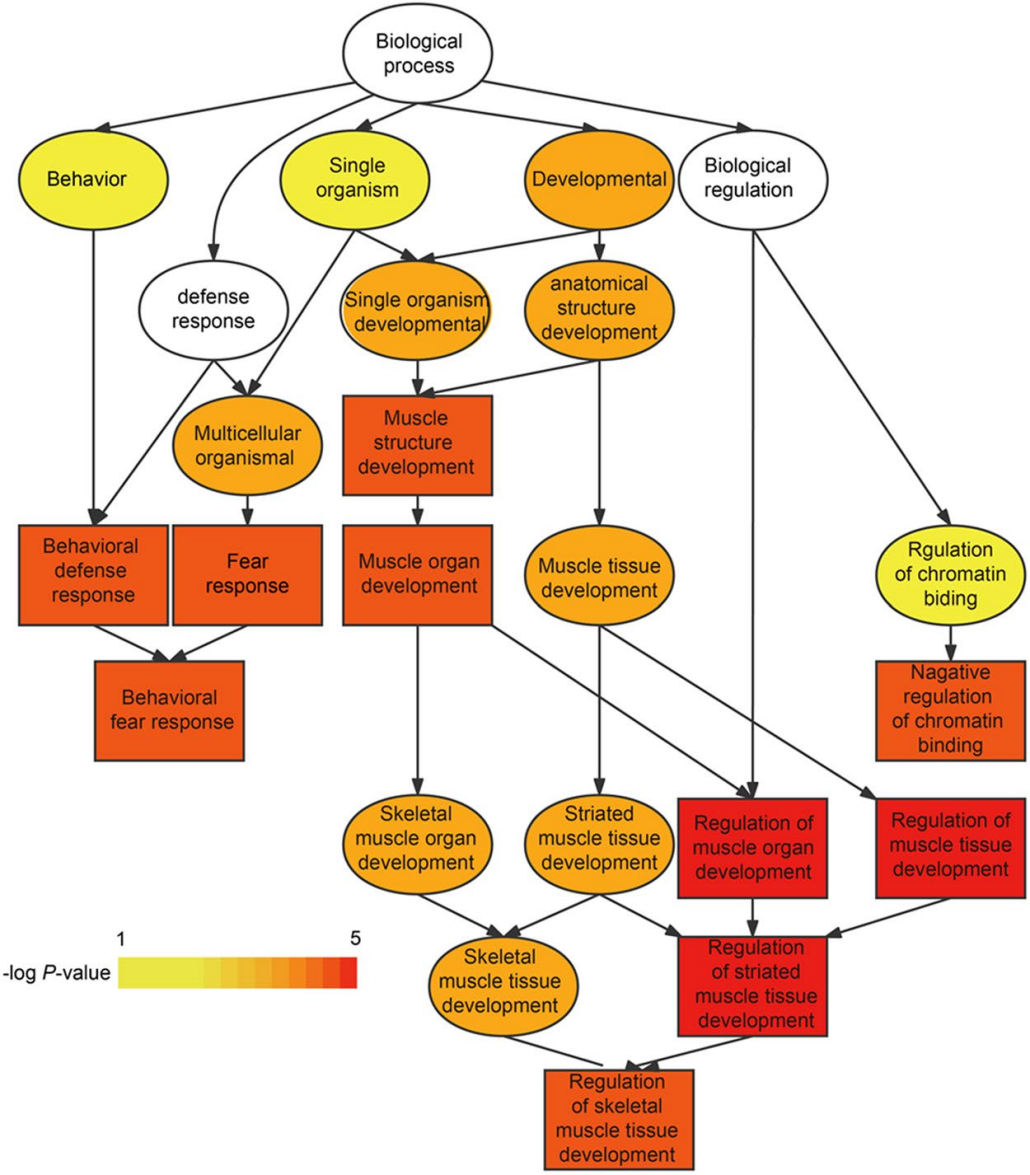
Functional cluster of top 10 GO terms enriched for AS genes specifically expressed in leg muscle. Colors from light to dark represent significance level of enriched GO terms (weak to strong).

Our DASE enrichment analysis identified GO terms involved in the functional maintenance of organs and to differences in physiological stages (Supplementary Data S3). We also found that AS genes might be involved in the regulation of organ function and development. For instance, many immune-related GO terms were found to be enriched for AS genes specifically expressed in spleen, such as immune system process, T cell differentiation involved in immune response, negative regulation of T cell differentiation, and immune response. This indicates AS genes specifically expressed in spleen may play important roles in spleen function. In addition, many GO terms related to material metabolism were enriched for AS genes specifically expressed in liver. Previous studies have shown that the liver is a crucial organ for material metabolism^35,36^, which supports the findings of this study. In leg muscle, the GO term of “regulation of skeletal muscle tissue development” (GO: 0048641) was significantly enriched for AS genes specifically expressed in spleen. These genes included *BBS5*, *NRG4*, *IP6K3*, *AMPD1*, and *DYSF* (Fig. 8 and Supplementary Table S9). These results suggest that AS genes specifically spliced in leg muscle might play a major role in regulating leg muscle development.

**Figure 9:**
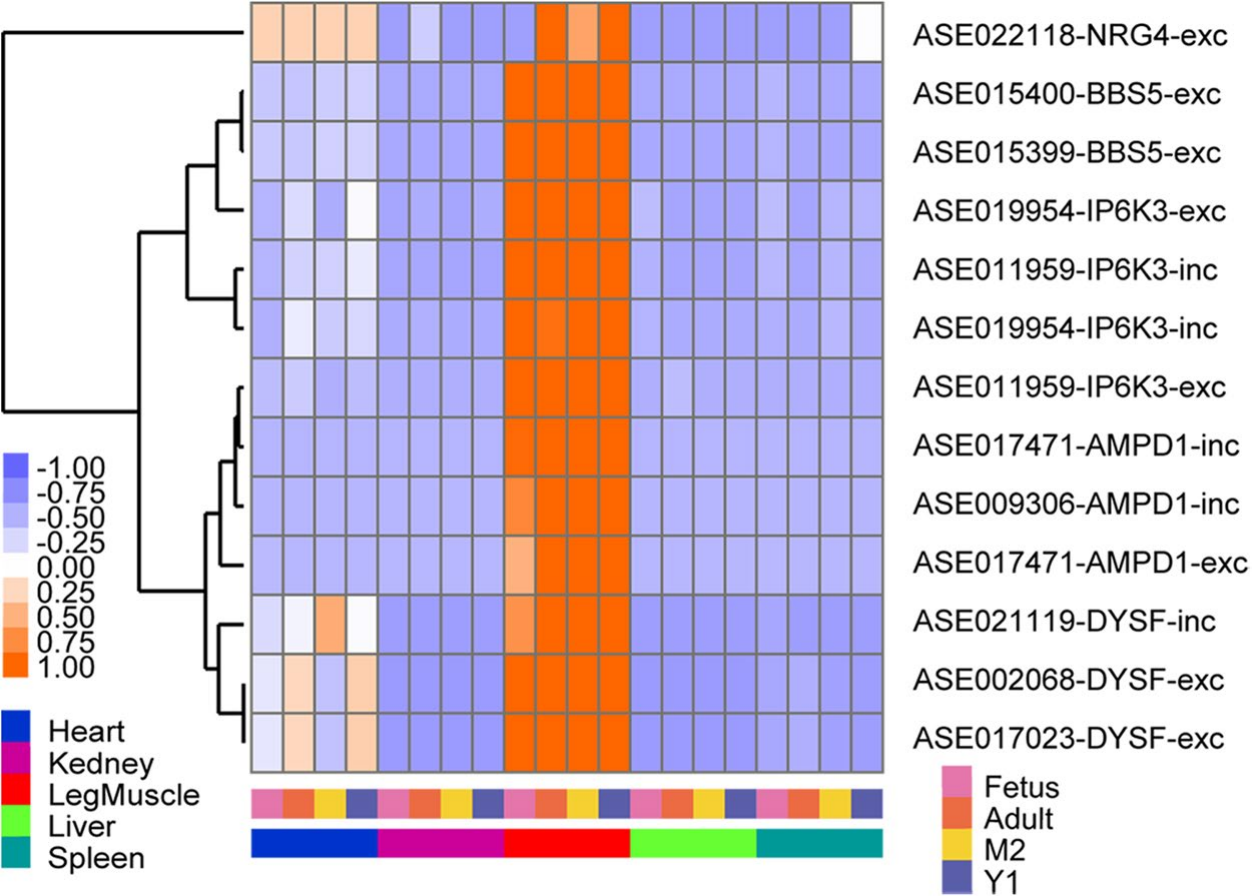
The 13 AS events, covered by *BBS5*, *NRG4*, *IP6K3*, *AMPD1*, and *DYSF* genes, were expressed higher in leg muscle than in other tissues.

To further investigate whether AS events might play key roles in leg muscle development, we analyzed the AS events covered by the five genes enriched in GO: 0048641 (Fig. 8 and Supplementary Table S9). In total, we detected 33 AS events in these five genes, of which 13 were specifically existed in leg muscle at significantly higher levels and one was existed in heart at significantly higher levels. The other 19 AS events were not significantly different in any tissues (Fig. 9 and Supplementary Table S9). Previous studies illustrated that four of these five genes played crucial roles in skeletal muscle development. *NRG4* (Neuregulin 4) has been shown to stimulate both the PI3K/AKT and STAT5 signaling pathway both *in vitro* and *in vivo*^37^. PI3K/Akt signaling plays important roles during *IGF1* promoted myoblast proliferation and skeletal muscle growth in embryonic chickens^38^. In addition, *STAT5*, which is required in GH (such as *IGF1*) actions^39^, is involved in animal skeletal muscle development. *DYSF* (dysferlin gene) plays a key role in muscle development, as evidenced by the role *DYSF* mutations play in human muscular dystrophy^40^ and that *DYSF* loss delays human muscle differentiation^41^. *AMPD1* (Adenosine Monophosphate Deaminase 1) has been identified as a candidate gene associated with meat production traits^42^, and a recent report revealed that *IP6K3* (inositol hexakisphosphate kinase 3) acts as an energy sensor^43^ and is involved in apoptosis^44^, and thus contributes to skeletal development. Taken together, we concluded that the 13 AS events differentially existed in leg muscle at significantly higher levels across these five genes are likely responsible for goat leg muscle development. However, further investigation is required to identify the mechanism through which these five genes and the 13 AS events regulate goat leg muscle development.

### Prediction of transcripts of unknown coding potential (TUCP)

**Figure 10:**
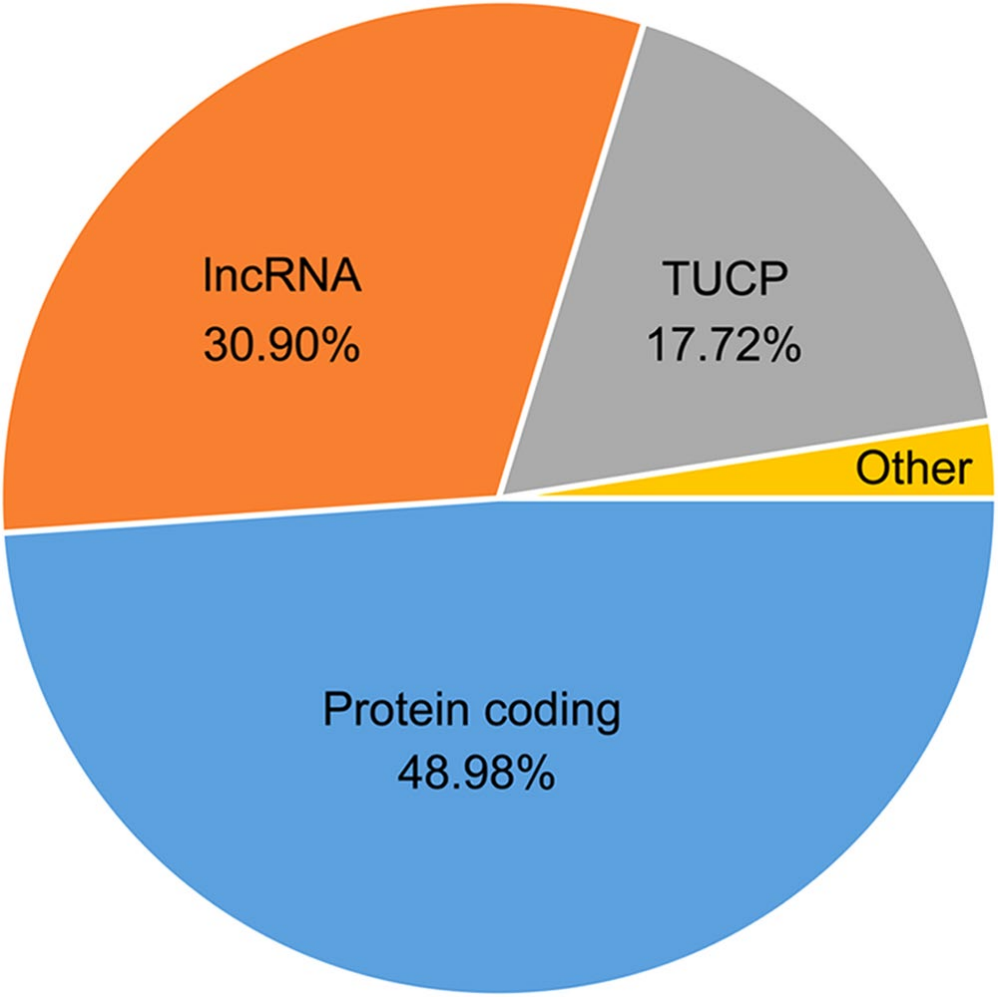
The prediction of transcripts of unknown coding potential (TUCP) for AS transcripts.

To investigate the coding potential of transcripts containing at least one AS event (AS transcripts), we performed prediction of transcripts of unknown coding potential (TUCP) (Fig. 10). We identified 30,160 transcripts (48.98% of total AS transcripts) with open reading frames that can be translated into proteins. In addition, 19,027 AS transcripts (30.90% of total AS transcripts) were identified as long non-coding RNA (lncRNA) and 10,915 transcripts (17.72% of total AS transcripts) were TUCP, which likely function as regulators of gene expression or protein function. The remained transcripts were considered as other transcripts in this study.

## Discussion

Since the AS phenomenon was first identified in the infectious adenovirus cycle^2,3^, it has been demonstrated that AS represents a fundamental regulatory process in all higher eukaryotes^5^. Because of this, AS has become an important research focus in the field of eukaryotic gene regulation^14^. In this study, we performed RNA-seq analysis to unearth the goat AS landscape for the first time. Our results indicate that 85.53% of intron-containing goat genes undergo AS, with an average of 2.72 AS events per gene (22,970 AS events/8,460 AS genes). The percentage of AS genes identified in this study is much higher than that of soybean (63%)^45^ but slightly lower than that in humans (95%)^12^. These results represent an underestimation of goat AS genes due to the fact that only samples from goats reared under normal conditions without external stresses were utilized in this study. This is in line with previous studies indicating many AS events under stress treatment^46^.

We found some outstanding features of goat introns compared to those of plants and other mammals. The average length of goat introns (6,103.75 nt) is much longer than other animals (chicken, lizard, and frog, average 3085.04 nt, 3974.93 nt, and 2161.58 nt, respectively) and plants, like *Arabidopsis* (average 298 nt)^13^. However, intron sizes of goat is much shorter than that of human. Surprisingly, we found 4,515 enormous introns (>50 kb) in our study (Supplementary Table S4), which is much more than previously observed in other mammals such as humans (3,473), mice (2,435), dogs (2,223) and chickens (853)^47^. Shepard *et al*.^47^ demonstrated that abundant amounts of repetitive elements (mainly SINEs and LINEs) in large introns can form stems with each other, and these stems with long loops within large introns allow intron splice sites to quickly identify one another, reducing the distance between donor and acceptor sites. Therefore, there should be more functional repetitive elements in the goat genome than other mammalian genomes, which will stimulate future research into goat intron splicing.

RI AS events were the most common AS type identified in this study (37.04%). This is contradictory to previous results in human^48–50^ where RI AS events have been found to be much less frequent. However, a recent study has indicated that RI AS events are far more frequent in mammals than previously predicted, and that ~53% and ~51% of all human and mouse introns have the potential to be retained in poly(A)+ transcripts, respectively^51^. Thus, the high RI percentage observed in the goat genome is well supported, and warrants further investigation in the future.

Previous observations have indicated many AS events are tissue-specific^45^. Our study demonstrated that the number and frequency of AS events vary dramatically in the different tissues (Supplementary Data S2). The number of AS events identified was higher in functionally complex tissues, such as spleen and kidney. This result is consistent with previous reports in nervous system and brain^33,52^. Recent studies have demonstrated that AS not only can increase proteome diversity, but also regulate gene expression^14,53^. Therefore, the tissue-specific AS events obtained in this study will provide a strong basis in further investigation of the effects of AS on proteome diversity and gene expression.

Previous analysis of AS events in human tissues indicated that skeletal muscle is one of the tissues with the highest expression of tissue specific alternative exons^50,54^. In this study, we identified 1,319 AS events specifically existed in goat leg muscle, many of which are involved in goat leg muscle development, including five genes (*BBS5*, *NRG4*, *IP6K3*, *AMPD1*, and *DYSF*) associated with the “regulation of skeletal muscle tissue development” GO term. 13 of the AS events harbored by these five genes were differentially existed in leg muscle at significantly higher levels. Four of the five genes (*NRG4*, *IP6K3*, *AMPD1*, and *DYSF*) have been previously reported to play crucial roles in skeletal muscle development. These results provide a basis in investigating the potential splicing-related regulatory mechanism of these five genes in goat leg muscle development.

In conclusion, we performed a comprehensive analysis of the goat AS landscape at the genome-wide level for the first time. These results provide a valuable resource for understanding gene expression and the biological function of AS in goats. Our data, which only contains the tissues in normal conditions, combined with stress-associated goat AS profiles will present tremendous resources for exploring the regulatory mechanisms underlying goat tissue development.

## Materials and Methods

### Animal management

The management of Hainan Black goats used in this study was identical with that described in our previous work^55^. Briefly, animals were reared on cultivated grasses including king grass (*Pennisetum purpureum K*. *Scbumacb* × *P*. *typhoideum Rich*), stylo (*Stylosanthes guianensias SW*.), *paspalum* (*Paspalum scrobiculatum Linn*.) and guinea grass (*Panicum maximum Jacq*.). The goats received routine vaccinations to general epidemic diseases yearly in spring and autumn. All kids stayed with their mothers up to weaning at 2 months of age. Pre-weaning kids had free access to the cultured grasses ad libitum and were supplied with kids’ concentrated supplement. The post-weaning kids were separated from their mothers and penned together. Because the body weight of Hainan Black goats continuously increases till two years old of age, goats > two years of age were considered adult goats.

### Sample collection

Three healthy goats from each of four developmental stages were selected. The four developmental stages were embryonic late stage (the embryonic ages beyond 135 d, Fetus), two months of age (M2), one year of age (Y1), and adult (>two years old). Leg muscle, kidney, heart, liver, and spleen were collected from each goat at each developmental stage in sterile condition. All samples were collected within 15 min after exsanguination, immediately immersed in liquid nitrogen, and stored at −80 °C.

### RNA isolation, RNA-seq library preparation and sequencing

RNA isolation, RNA-seq library preparation and sequencing were performed as previously described^56^. Briefly, total RNA was isolated from all samples using the RNAiso plus kit (Takara, Dalian, China) following the manufacturer’s instructions. The RNA quality was analyzed by 1.0% agarose gel electrophoresis and spectrophotometric absorption at 260 nm in a Nanodrop ND-1000® Spectrophotometer. All RNA samples were treated with DNase I recombinant (Roche, Shanghai, China). The mRNA was separated from 6 mg of total RNA and fragmented into short fragments using fragmentation buffer. After first strand cDNA synthesis and purification, sequencing adapters were ligated to the 5′ and 3′ ends of the fragments, after which the products with 5′ and 3′ adapters were amplified purified. Finally, the libraries were sequenced on Illumina sequencing platform (HiSeq^TM^ 2500).

### The evaluation of RNA-seq data

We evaluated the reliability of our RNA-seq data in identifying and analyzing goat AS profiles based on two aspects. First, we assessed read distributions along goat annotated genes. Wang *et al*. (2009) revealed RNA fragmentation provides more even coverage along the gene body, while reducing coverage at the 5′ and 3′ ends during the RNA-seq library construction^57^. As this approach was used to construct our libraries, we assessed the read distribution along goat annotated genes. Overall, most reads mapped to the body of goat annotated genes for each sample (Supplementary Fig. S1a), which is consistent with Wang *et al*.^57^. We then performed coverage analysis of each annotated transcript to assess the percentage of each transcript covered. The results indicated that more than 80% of the annotated transcripts were covered by at least four uniquely mapped reads in our RNA-seq data (Supplementary Fig. S1b). Taken together, the results above indicate that our RNA-seq data is of high enough quality to comprehensively evaluate the goat AS landscape.

### Read alignment

First, we removed sequencing adaptors using Trim Galore version 0.3.7, which automatically identifies and removes adaptor sequences in paired-end reads. We also removed low quality sequences (reads where more than 30% of the bases had PHRED quality scores <20) and ambiguous bases using homemade Perl scripts. Then, we downloaded the goat genome sequence (build CHIR1.0) and gene annotation from NCBI (ftp://ftp.ncbi.nlm.nih.gov/genomes/Capra_hircus/). Next, we mapped the high-quality reads to the goat reference genome using TopHat2 version 2.2.6^26^, utilizing Bowtie2 version 2.1.0^58^ for mapping, allowing a maxim total alignment gap length of 3 nt (–read-gap-length 3), no more than 2 mismatches (–read-mismatches 2), and setting the –read-edit-dist option to 3. The read realign edit distance was set as 0, and no discordant reads pair were reported. The uniquely mapped reads were used for further analysis.

### Transcriptome assembly

To increase the transcriptome assembly accuracy, we first removed potential erroneous reads covering SJs or distributed in exonic regions. Specifically, we used TopHat2, which reduces the false discovery of introns in tandem repeats, to predict SJs following the method described in Marquez *et al*.^13^. To eliminate false positives resulting from erroneous alignments, we filtered out SJs predicted from reads with mismatches. We also filtered out SJs supported by one or more reads with mismatches that were within 10 nt of a junction supported by perfectly matched reads. Only SJs supported by at least 2 reads after removing PCR duplicates were considered in downstream analysis.

To investigate the transcription atlas of each tissue at each developmental stage, we merged the filtered reads of the three biological replicates and assembled a meta-transcriptome using Cufflinks2 version 2.2.1^59^, with the following parameters (−F 0.1 −j 0.15 −u −b) to reduce misidentification of anti-sense transcripts and incorrect fusions of two or more transcripts with the genome reference^14^. We used the genome sequence for read fragment bias correction, and set a threshold of 0.1 to filter out low abundance isoforms that may not be reliably assembled^60^. To reduce the false discovery of RI, we ignored incompletely spliced isoforms with a relative abundance cutoff of 0.15, which is calculated from the minimum coverage depth in the intronic region divided by the number of spliced reads^59^. After producing the 20 meta-assembles, we merged them using Cuffmerge version 2.2.1. We mapped assembled transcripts against all CHIR1.0 gene models using Cuffcompare version 2.2.1, which filters out redundant transcripts with the same intron chain but different transcription start or stop sites.

### Expression estimation of assembled transcript

Expression levels of the assembled transcripts were determined using Cufflinks version 2.2.1^59^. Normalized abundance estimates (FPKM) were computed for all assembled transcripts, by applying the geometric normalization method^61^.

### Identification of DASE

By providing the assembled gene models, we identified the main five types of AS events using rMATS^16^. To minimal the false discovery of AS events, we removed events within intergenic regions, and those with different transcription directions. We defined the exon exclusion isoform (EEI) as the transcript with a larger intron, and exon inclusion isoform (EII) as the transcript with a shorter intron. Then, we quantified and normalized each isoform by counting the reads spanning the spliced region in each sample. Taking the abundance of AS derived transcripts into account, we assessed the relative abundance of EII and EEI by defining AS score as follows:

1 $$AS\,score=\frac{PSI-0.5}{0.5}$$

2 $$PSI=\frac{Count{s}_{EII}}{Count{s}_{EII}+Count{s}_{EEI}}$$

AS-score is transformed from percent spliced in (PSI), which is defined by Burge lab^62^ and also employed by rMATS^16^. AS-score varies from −1 to 1, with −1 ony only EEI expression, 1 indicating only EEI expression, and 0 indicating equal expression of both isoforms. If the AS score ranged from 0 to1, it indicates the EII form is the major isoform.

The DASE were identified by comparing samples across different developmental stages within the same tissue, and samples across different tissues within the same developmental stage using the likelyhood-ratio test with three biological replicates. AS events with a false discovery rate (FDR) <0.05 were considered differentially AS events.

### Identification of tissue- or developmental stage- specific AS events

To identify tissue or developmental stage- specific AS events, we investigated the expression- specificity of each AS transcript following the pipeline of Supplementary Fig. S2. Because one pre-mRNA could be alternative spliced into different isoforms, we used rMATS to quantify the normalized local read count of ESI and EII for each AS event instead of quantifying the global abundance of the whole transcript. To reduce the false discovery rate, we filtered out isoforms with low (read count <3) coverage. Then, we used Tau (τ) to measure the specificity. Tau is one of the most robust methods^34^, taking both expression abundance and the number of samples into consideration. It is defined as:

3 $$\tau =\frac{{\sum }_{i=1}^{n}(1-\widehat{{x}_{i}})}{n-1\,};\,\widehat{{x}_{i}}=\frac{{x}_{i}}{{{\rm{\max }}}_{1\le i\le n}({x}_{i})}$$

If one isoform was expressed in a single tissue or developmental stage, it was considered a tissue- or developmental stage- specific AS event, respectively.

### Gene functional analysis

As many goat genes do not have GO annotations, we first performed diamond blastx^63^ search all goat annotated cDNAs obtained from CHIR 1.0 against NCBI non-redundant protein database (nr; ftp://ftp.ncbi.nih.gov/blast/db/FASTA/) with a cutoff e-value of 10^−5^. Sequences were further analyzed by Blast 2GO version 2.5^64^ with the default parameters using updated databases for GO (gene ontology) mapping, inter-pro-scan, enzyme code. In addition, we used all the expressed genes detected in this study as the background to perform GO enrichment analysis of specific or differential expressed genes. During the GO analysis, we used hypergeometric test, implemented in topGO^65^, and adjusted the P-values by FDR.

### Prediction of transcripts of unknown coding potential (TUCP)

We followed the methods described in Lyer *et al*.^66^, which integrated predictions from the alignment-free Coding Potential Assessment Tool (CPAT)^67^ and searched for Pfam 30.0^68^ matches to assess the coding potential. CPAT uses a logistic regression model and takes four sequence features as parameters: open reading frame size, open reading frame coverage, Fickett TESTCODE statistic, and hexamer usage bias. To optimize the balanced accuracy metric, we randomly sampled 2,000 of the putative noncoding and protein-coding transcripts. Finally, we ued a CPAT probability of 0.40 as the cutoff, as it achieved accurate discrimination of lncRNAs and protein-coding genes (sensitivity = 0.97, specificity = 0.97, Supplementary Fig. S3). As additional evidence of coding potential, we scanned all transcripts for Pfam A or B domains across the six reading frames. We designated putative noncoding transcripts with either a Pfam domain or a positive CPAT prediction as TUCPs.

### Ethics statement

All sample collection and subsequent experiments were approved by, and all methods were performed in accordance with, the Ethical and Animal Welfare Committee of Beijing, China. Goats were slaughtered using the electric shock method followed by jugular vein bloodletting method within 30 seconds to ameliorate their suffering.

## Electronic supplementary material

Supplementary Information

Supplementary Table S4

Supplementary Table S6

Dataset 1

Dataset 2

Dataset 3

## Data Availability

RNA-seq data used in this study has been uploaded to the Short Read Archive (SRA) under the accession number SRP109247. In addition, we have built a database to display and easily download our results (http://xufeng.ngrok.xiaomiqiu.cn/jbrowser/JBrowse-1.12.3/index.html?data=goat&loc=NC_005044.2%3A1206.1408&tracks=goat_AS_gtf%2CTotal.bed%2CGenes.bed&highlight=).
