# Supplementary material for: Landscape of alternative splicing in Capra_hircus
Source: Sci Rep. 2018 Oct 11;8:15128. doi: 10.1038/s41598-018-33078-7 (PMC6181917; doi:10.1038/s41598-018-33078-7)
Supplement: Supplementary file 1 — Supplementary Information [file 41598_2018_33078_MOESM1_ESM.doc]

**Landscape of alternative splicing in *Capra_hircus***

Tieshan Xu^1,†^, Feng Xu^3,†^, Lihong Gu^2,†^, Guang Rong^1^, Mao Li^1^, Fei Qiao^1^ , Liguang Shi1, Dingfa Wang^1^, Wanliang Xia^1^, Wenjuan Xun^1^, Ting Cao^1^, Yiming Liu^1^, Zhemin Lin^2,*^, Hanlin Zhou^1,*^

^1^ Tropical Crop Genetic Resource Research Institute, Chinese Academy of Tropical Agricultural Sciences, Danzhou, Hainan, P.R. China

^2^ Institute of Animal Science & Veterinary, Hainan Academy of Agricultural Science, Haikou, P.R. China

^3^ National Maize Improvement Center, China Agricultural University, Beijing, P.R. China

* Correspondence and requests for materials should be addressed to H.Z. (Email: [zhouhanlin8@163.com](mailto:zhouhanlin8@163.com)) or to Z.L. (Email: linzhemin2017@163.com)

^†^These authors contributed equally to this work.

In this study, there are some supplementary files those do not include in the main manuscript text. The files include three Supplementary datasets (Supplementary dataset S1-S3), three Supplementary Figure (Supplementary Figure S1-S3), and nine Supplementary Tables (Supplementary Table S1-S9). Among which, because Supplementary dataset S1-S3, and Supplementary Table S4 and S6 are very large, these files were included as separate Excel documents and were uploaded separately from the main article file and supplementary information. The legends of supplementary files and Supplementary Table S1-S3, S5, S7-S9 and Supplementary Fig. S1-S3 were listed as follow.

**The legends of supplementary files**

Supplementary Dataset S1. Tissue- specific AS events (xlsx)

Supplementary Dataset S2. Developmental stage- specific AS events (xlsx)

Supplementary Dataset S3. Significantly enriched GO terms of AS genes specifically expressed in different tissues (xlsx)

Supplementary Figure S1. **Reliability evaluation of RNA-seq data for identifying and analyzing the goat AS landscape**. (**a**) The distribution of mapped reads along goat annotated genes. (**b**) The percentage of goat annotated transcripts covered by at least four uniquely mapped reads.

Supplementary Figure S2. The pipeline for identification of tissue- or developmental stage- specific AS events

Supplementary Figure S3. The sensitivity and specificity in discrimination of lncRNAs and protein-coding genes using CPAT with a 0.40 cutoff. (A) ROC curve. (B) PR (precision-recall) curve. (C) Accuracy vs cutoff value. (D) Two graphic ROC curve to determine the optimum cutoff value.

Supplementary Table S1. Samples from different developmental stages for RNA-seq used in this study (docx)

Supplementary Table S2. The basic information of sequenced data (docx)

Supplementary Table S3. The statistics of reads alignment along the goat genome (docx)

Supplementary Table S4. Characteristics of obtained introns in this study (xlsx)

Supplementary Table S5. AU-richness and intron-size of six other species (docx)

Supplementary Table S6. AS events detected in this study (xlsx)

Supplementary Table S7. Differential alternative splicing events (DASE) among tissues at the same stage (docx)

Supplementary Table S8. DASE among developmental stages for the same tissue

Supplementary Table S9. The detailed information of 5 genes likely functioned in leg muscle development (docx)

**Supplementary Table S1-S3, S5, S7 and Supplementary Fig. S1-S3**

Table……..................................................................................................................... 3-17

Table S1 ..................................................................... ................................................. 3

Table S2 ....................................................................................................................... 4-5

Table S3 ................................................................................................................ ...... 6-7

Table S5 ............................................................................ ............................. ....... ...... 8

Table S7....................................................................................................................... 9-10

Table S8....................................................................................................................... 11-14

Table S9 .......................................................................................................................15-17

Figure…… ................................................................................................................... 18-20

Fig. S1 .......................................................................................................................... 18

Fig. S2 .......................................................................................................................... 19

Fig. S3 .......................................................................................................................... 20

Supplementary Table S1. Samples from different developmental stages for RNA-seq used in this study

| Stages | Samples | Replicates |
| --- | --- | --- |
| Fetus (the fetuses with their embryonic ages beyond 135 d) | Heart | 3 |
|  | Kidney | 3 |
|  | Leg Muscle | 3 |
|  | Liver | 3 |
|  | Spleen | 3 |
| M2 (two months of age) | Heart | 3 |
|  | Kidney | 3 |
|  | Leg Muscle | 3 |
|  | Liver | 3 |
|  | Spleen | 3 |
| Y1 (one year of age) | Heart | 3 |
|  | Kidney | 3 |
|  | Leg Muscle | 3 |
|  | Liver | 3 |
|  | Spleen | 3 |
| Adult (the goats with their age beyond two years) | Heart | 3 |
|  | Kidney | 3 |
|  | Leg Muscle | 3 |
|  | Liver | 3 |
|  | Spleen | 3 |

Supplementary Table S2. The basic information of sequenced data

| Sample | Raw Reads | Clean reads | Raw bases(G) | Clean bases(G) | Error  (%) | Q20  (%) | GC  (%) |
| --- | --- | --- | --- | --- | --- | --- | --- |
| Heart_Fetus_R1 | 21431300 | 21059720 | 5.36 | 5.26 | 0.04 | 94.98 | 49.87 |
| Heart_Fetus_R2 | 20497601 | 20110496 | 5.12 | 5.03 | 0.04 | 94.46 | 51.02 |
| Heart_Fetus_R3 | 23529177 | 23075653 | 5.88 | 5.77 | 0.04 | 94.94 | 48.26 |
| Heart_M2_R1 | 26612040 | 26169203 | 6.65 | 6.54 | 0.04 | 95.65 | 47.67 |
| Heart_M2_R2 | 22940106 | 22611470 | 5.74 | 5.65 | 0.04 | 95.62 | 48.29 |
| Heart_M2_R3 | 22758191 | 22289143 | 5.69 | 5.57 | 0.04 | 95.53 | 47.69 |
| Heart_Y1_R1 | 25746842 | 25386350 | 6.44 | 6.35 | 0.04 | 95.66 | 49.16 |
| Heart_Y1_R2 | 23986798 | 23587104 | 6.00 | 5.90 | 0.04 | 95.84 | 48.73 |
| Heart_Y1_R3 | 20885173 | 20549387 | 5.22 | 5.14 | 0.03 | 95.89 | 47.07 |
| Heart_Adult_R1 | 29752317 | 29060555 | 7.44 | 7.27 | 0.05 | 92.78 | 50.20 |
| Heart_Adult_R2 | 22730677 | 22241334 | 5.68 | 5.56 | 0.04 | 93.05 | 49.06 |
| Heart_Adult_R3 | 24955726 | 24370596 | 6.24 | 6.09 | 0.05 | 92.93 | 49.22 |
| Kidney_Fetus_R1 | 24708629 | 24262122 | 6.18 | 6.07 | 0.04 | 94.60 | 49.93 |
| Kidney_Fetus_R2 | 25459236 | 24902788 | 6.36 | 6.23 | 0.04 | 94.59 | 48.92 |
| Kidney_Fetus_R3 | 25570070 | 25069044 | 6.39 | 6.27 | 0.04 | 94.82 | 51.18 |
| Kidney_M2_R1 | 23593293 | 23191957 | 5.90 | 5.80 | 0.04 | 94.30 | 52.59 |
| Kidney_M2_R2 | 23816793 | 23185129 | 5.95 | 5.80 | 0.04 | 94.33 | 52.75 |
| Kidney_M2_R3 | 22910190 | 22510143 | 5.73 | 5.63 | 0.04 | 94.57 | 50.75 |
| Kidney_Y1_R1 | 24563345 | 24116402 | 6.14 | 6.03 | 0.04 | 94.22 | 51.18 |
| Kidney_Y1_R2 | 31962886 | 31331729 | 7.99 | 7.83 | 0.04 | 94.21 | 50.85 |
| Kidney_Y1_R3 | 29085810 | 28492093 | 7.27 | 7.12 | 0.04 | 94.19 | 51.25 |
| Kidney_Adult_R1 | 25510677 | 25037587 | 6.38 | 6.26 | 0.04 | 93.39 | 48.25 |
| Kidney_Adult_R2 | 23927353 | 23480956 | 5.98 | 5.87 | 0.04 | 93.48 | 47.19 |
| Kidney_Adult_R3 | 23899609 | 23431986 | 5.97 | 5.86 | 0.04 | 93.43 | 47.89 |
| LegMuscle_Fetus_R1 | 22094520 | 21688234 | 5.52 | 5.42 | 0.04 | 94.09 | 49.91 |
| LegMuscle_Fetus_R2 | 20314897 | 19958088 | 5.08 | 4.99 | 0.04 | 95.47 | 51.44 |
| LegMuscle_Feus_R3 | 19252269 | 18895288 | 4.81 | 4.72 | 0.04 | 94.86 | 51.34 |
| LegMuscle_M2_R1 | 18923368 | 18501360 | 4.73 | 4.63 | 0.04 | 94.55 | 51.55 |
| LegMuscle_M2_R2 | 21794788 | 21370541 | 5.45 | 5.34 | 0.04 | 94.49 | 51.57 |
| LegMuscle_M2_R3 | 22188739 | 21713134 | 5.55 | 5.43 | 0.04 | 94.47 | 51.36 |
| LegMuscle_Y1_R1 | 20860345 | 20473210 | 5.22 | 5.12 | 0.04 | 94.29 | 50.99 |
| LegMuscle_Y1_R2 | 20733544 | 20355868 | 5.18 | 5.09 | 0.04 | 94.47 | 51.96 |
| LegMuscle_Y1_R3 | 22755490 | 22361172 | 5.69 | 5.59 | 0.04 | 94.58 | 49.96 |
| LegMuscle_Adult_R1 | 19762762 | 19393959 | 4.94 | 4.85 | 0.04 | 94.12 | 50.81 |
| LegMuscle_Adult_R2 | 19635091 | 19214973 | 4.91 | 4.80 | 0.04 | 93.85 | 51.20 |
| LegMuscle_Ault_R3 | 20516269 | 20101361 | 5.13 | 5.03 | 0.04 | 94.66 | 51.52 |
| Liver_Fetus_R1 | 23622620 | 23227498 | 5.91 | 5.81 | 0.04 | 94.65 | 49.69 |
| Liver_Fetus_R2 | 24126660 | 23735371 | 6.03 | 5.93 | 0.04 | 95.00 | 48.83 |
| Liver_Fetus_R3 | 25719013 | 25358333 | 6.43 | 6.34 | 0.04 | 95.06 | 48.67 |
| Liver_M2_R1 | 18332597 | 18243800 | 4.58 | 4.56 | 0.04 | 94.59 | 48.52 |
| Liver_M2_R2 | 21265596 | 21169946 | 5.32 | 5.29 | 0.04 | 94.74 | 48.61 |
| Liver_M2_R3 | 19570192 | 19474488 | 4.89 | 4.87 | 0.04 | 94.77 | 49.23 |
| Liver_Y1_R1 | 21537332 | 21277721 | 5.38 | 5.32 | 0.04 | 95.25 | 48.38 |
| Liver_Y1_R2 | 21539620 | 21252942 | 5.38 | 5.31 | 0.04 | 95.19 | 48.42 |
| Liver_Y1_R3 | 21831871 | 21547060 | 5.46 | 5.39 | 0.04 | 95.22 | 48.01 |
| Liver_Adult_R1 | 21536177 | 21390786 | 5.38 | 5.35 | 0.04 | 94.81 | 49.29 |
| Liver_Adult_R2 | 20386929 | 20232544 | 5.10 | 5.06 | 0.04 | 94.64 | 48.49 |
| Liver_Adult_R3 | 25668932 | 25476258 | 6.42 | 6.37 | 0.04 | 94.82 | 49.90 |
| Spleen_Fetus_R1 | 22131055 | 21807248 | 5.53 | 5.45 | 0.04 | 95.34 | 49.08 |
| Spleen_Fetus_R2 | 23232658 | 22894832 | 5.81 | 5.72 | 0.04 | 95.41 | 48.86 |
| Spleen_Fetus_R3 | 27768136 | 27336127 | 6.94 | 6.83 | 0.04 | 95.67 | 48.77 |
| Spleen_M2_R1 | 21593749 | 21148617 | 5.40 | 5.29 | 0.04 | 94.88 | 50.18 |
| Spleen_M2_R2 | 21342694 | 20975167 | 5.34 | 5.24 | 0.04 | 94.84 | 50.45 |
| Spleen_M2_R3 | 21260042 | 20909478 | 5.32 | 5.23 | 0.04 | 94.78 | 49.97 |
| Spleen_Y1_R1 | 19723967 | 19447526 | 4.93 | 4.86 | 0.04 | 95.27 | 49.83 |
| Spleen_Y1_R2 | 22090415 | 21690680 | 5.52 | 5.42 | 0.04 | 94.87 | 50.03 |
| Spleen_Y1_R3 | 21150976 | 20813643 | 5.29 | 5.20 | 0.04 | 94.84 | 49.55 |
| Spleen_Adult_R1 | 22885548 | 22530615 | 5.72 | 5.63 | 0.04 | 95.39 | 50.44 |
| Spleen_Adult_R2 | 23439563 | 23100597 | 5.86 | 5.78 | 0.04 | 95.28 | 51.46 |
| Spleen_Adult_R3 | 25246082 | 24878458 | 6.31 | 6.22 | 0.04 | 95.17 | 49.88 |
| Average | 22944472 | 22557831 | 5.74 | 5.64 | 0.04 | 94.70 | 49.79 |
| Total | 1.377E+09 | 1.353E+09 | 344.16 | 338.38 |  |  |  |

Supplementary Table S3. The statistics of reads alignment along the goat genome

| Sample | Clean reads | Mapped reads | Mapping ratio | The statistics of mapped reads | | | |
| --- | --- | --- | --- | --- | --- | --- | --- |
|  |  |  |  | Multiplely mapped reads | Uniquely mapped reads | Multiplely mapped ratio | Uniquely mapped ratio |
| Heart_Fetus_R1 | 19,762,206 | 15,283,980 | 77.34% | 411,744 | 14,872,236 | 2.69% | 97.31% |
| Heart_Fetus_R2 | 18,698,378 | 14,068,472 | 75.24% | 381,783 | 13,686,689 | 2.71% | 97.29% |
| Heart_Fetus_R3 | 21,681,593 | 17,422,005 | 80.35% | 734,412 | 16,687,593 | 4.22% | 95.78% |
| Heart_M2_R1 | 24,889,718 | 19,985,016 | 80.29% | 742,177 | 19,242,839 | 3.71% | 96.29% |
| Heart_M2_R2 | 21,426,811 | 17,602,264 | 82.15% | 643,757 | 16,958,507 | 3.66% | 96.34% |
| Heart_M2_R3 | 21,131,051 | 16,958,047 | 80.25% | 831,937 | 16,126,110 | 4.91% | 95.09% |
| Heart_Y1_R1 | 24,042,789 | 19,750,334 | 82.15% | 628,119 | 19,122,215 | 3.18% | 96.82% |
| Heart_Y1_R2 | 22,504,818 | 17,738,319 | 78.82% | 674,980 | 17,063,339 | 3.81% | 96.19% |
| Heart_Y1_R3 | 19,607,053 | 16,270,911 | 82.98% | 664,304 | 15,606,607 | 4.08% | 95.92% |
| Heart_Adult_R1 | 26,062,134 | 19,588,538 | 75.16% | 643,933 | 18,944,605 | 3.29% | 96.71% |
| Heart_Adult_R2 | 20,070,482 | 15,228,196 | 75.87% | 512,023 | 14,716,173 | 3.36% | 96.64% |
| Heart_Adult_R3 | 21,938,145 | 16,905,041 | 77.06% | 601,067 | 16,303,974 | 3.56% | 96.44% |
| Kidney_Fetus_R1 | 22,484,414 | 18,024,389 | 80.16% | 317,942 | 17,706,447 | 1.76% | 98.24% |
| Kidney_Fetus_R2 | 23,040,733 | 18,574,352 | 80.62% | 407,898 | 18,166,454 | 2.20% | 97.80% |
| Kidney_Fetus_R3 | 23,436,848 | 18,314,480 | 78.14% | 337,735 | 17,976,745 | 1.84% | 98.16% |
| Kidney_M2_R1 | 21,617,833 | 15,933,390 | 73.70% | 314,507 | 15,618,883 | 1.97% | 98.03% |
| Kidney_M2_R2 | 21,590,317 | 14,803,138 | 68.56% | 303,129 | 14,500,009 | 2.05% | 97.95% |
| Kidney_M2_R3 | 20,989,777 | 15,552,214 | 74.09% | 272,774 | 15,279,440 | 1.75% | 98.25% |
| Kidney_Y1_R1 | 22,356,246 | 16,732,418 | 74.84% | 362,261 | 16,370,157 | 2.17% | 97.83% |
| Kidney_Y1_R2 | 29,065,005 | 21,460,367 | 73.84% | 427,494 | 21,032,873 | 1.99% | 98.01% |
| Kidney_Y1_R3 | 26,473,181 | 19,512,033 | 73.70% | 423,371 | 19,088,662 | 2.17% | 97.83% |
| Kidney_Adult_R1 | 22,564,313 | 18,712,848 | 82.93% | 467,537 | 18,245,311 | 2.50% | 97.50% |
| Kidney_Adult_R2 | 21,183,546 | 17,710,753 | 83.61% | 407,843 | 17,302,910 | 2.30% | 97.70% |
| Kidney_Adult_R3 | 21,122,260 | 17,676,926 | 83.69% | 416,758 | 17,260,168 | 2.36% | 97.64% |
| LegMuscle_Fetus_R1 | 19,939,133 | 16,217,723 | 81.34% | 487,198 | 15,730,525 | 3.00% | 97.00% |
| LegMuscle_Fetus_R2 | 18,906,246 | 15,101,887 | 79.88% | 366,870 | 14,735,017 | 2.43% | 97.57% |
| LegMuscle_Fetus_R3 | 17,693,011 | 14,237,994 | 80.47% | 384,260 | 13,853,734 | 2.70% | 97.30% |
| LegMuscle_M2_R1 | 17,308,126 | 14,012,540 | 80.96% | 427,040 | 13,585,500 | 3.05% | 96.95% |
| LegMuscle_M2_R2 | 19,851,404 | 16,308,790 | 82.15% | 465,707 | 15,843,083 | 2.86% | 97.14% |
| LegMuscle_M2_R3 | 20,163,557 | 16,288,385 | 80.78% | 446,867 | 15,841,518 | 2.74% | 97.26% |
| LegMuscle_Y1_R1 | 18,942,069 | 15,769,817 | 83.25% | 426,186 | 15,343,631 | 2.70% | 97.30% |
| LegMuscle_Y1_R2 | 18,933,625 | 15,415,544 | 81.42% | 458,613 | 14,956,931 | 2.98% | 97.02% |
| LegMuscle_Y1_R3 | 20,795,249 | 17,535,038 | 84.32% | 528,461 | 17,006,577 | 3.01% | 96.99% |
| LegMuscle_Adult_R1 | 17,862,851 | 14,766,035 | 82.66% | 439,229 | 14,326,806 | 2.97% | 97.03% |
| LegMuscle_Adult_R2 | 17,555,843 | 14,337,248 | 81.67% | 355,120 | 13,982,128 | 2.48% | 97.52% |
| LegMuscle_Adult_R3 | 18,683,175 | 15,265,528 | 81.71% | 452,321 | 14,813,207 | 2.96% | 97.04% |
| Liver_Fetus_R1 | 21,533,370 | 17,334,141 | 80.50% | 316,885 | 17,017,256 | 1.83% | 98.17% |
| Liver_Fetus_R2 | 22,177,338 | 18,527,148 | 83.54% | 326,248 | 18,200,900 | 1.76% | 98.24% |
| Liver_Fetus_R3 | 23,751,053 | 19,683,820 | 82.88% | 344,604 | 19,339,216 | 1.75% | 98.25% |
| Liver_M2_R1 | 21,345,155 | 17,597,807 | 82.44% | 386,925 | 17,210,882 | 2.20% | 97.80% |
| Liver_M2_R2 | 20,856,254 | 17,039,043 | 81.70% | 388,127 | 16,650,916 | 2.28% | 97.72% |
| Liver_M2_R3 | 19,190,526 | 15,513,087 | 80.84% | 309,022 | 15,204,065 | 1.99% | 98.01% |
| Liver_Y1_R1 | 20,026,821 | 16,323,856 | 81.51% | 312,021 | 16,011,835 | 1.91% | 98.09% |
| Liver_Y1_R2 | 19,992,108 | 16,248,203 | 81.27% | 311,027 | 15,937,176 | 1.91% | 98.09% |
| Liver_Y1_R3 | 20,227,065 | 16,550,774 | 81.82% | 326,426 | 16,224,348 | 1.97% | 98.03% |
| Liver_Adult_R1 | 21,128,470 | 16,195,271 | 76.65% | 313,877 | 15,881,394 | 1.94% | 98.06% |
| Liver_Adult_R2 | 19,981,123 | 15,921,070 | 79.68% | 334,312 | 15,586,758 | 2.10% | 97.90% |
| Liver_Adult_R3 | 25,180,898 | 19,021,275 | 75.54% | 385,546 | 18,635,729 | 2.03% | 97.97% |
| Spleen_Fetus_R1 | 20,516,405 | 16,399,680 | 79.93% | 340,567 | 16,059,113 | 2.08% | 97.92% |
| Spleen_Fetus_R2 | 21,540,192 | 17,198,350 | 79.84% | 345,155 | 16,853,195 | 2.01% | 97.99% |
| Spleen_Fetus_R3 | 25,941,139 | 21,396,176 | 82.48% | 436,207 | 20,959,969 | 2.04% | 97.96% |
| Spleen_M2_R1 | 19,946,754 | 15,382,551 | 77.12% | 441,191 | 14,941,360 | 2.87% | 97.13% |
| Spleen_M2_R2 | 19,591,094 | 14,556,635 | 74.30% | 653,605 | 13,903,030 | 4.49% | 95.51% |
| Spleen_M2_R3 | 19,515,774 | 14,453,463 | 74.06% | 682,203 | 13,771,260 | 4.72% | 95.28% |
| Spleen_Y1_R1 | 18,276,615 | 13,960,869 | 76.39% | 686,286 | 13,274,583 | 4.92% | 95.08% |
| Spleen_Y1_R2 | 20,294,224 | 15,169,603 | 74.75% | 725,765 | 14,443,838 | 4.78% | 95.22% |
| Spleen_Y1_R3 | 19,417,008 | 15,195,880 | 78.26% | 668,175 | 14,527,705 | 4.40% | 95.60% |
| Spleen_Adult_R1 | 21,279,222 | 15,577,857 | 73.21% | 761,039 | 14,816,818 | 4.89% | 95.11% |
| Spleen_Adult_R2 | 21,757,758 | 15,427,717 | 70.91% | 911,537 | 14,516,180 | 5.91% | 94.09% |
| Spleen_Adult_R3 | 23,325,709 | 17,052,293 | 73.11% | 784,396 | 16,267,897 | 4.60% | 95.40% |
| Average | 21,186,100 | 16,713,192 | 78.98% | 477,642 | 16,235,550 | 2.86% | 97.14% |

Supplementary Table S5. AU-richness and intron-size of six other species

| Species | Items | AU_richness | Intron_size |
| --- | --- | --- | --- |
| Human | Average | 51.63% | 8527.22 |
|  | Median | 53.30% | 1873.00 |
| Mouse | Average | 53.55% | 6149.44 |
|  | Median | 55.10% | 1471.00 |
| Chicken | Average | 54.28% | 3085.04 |
|  | Median | 57.90% | 765.00 |
| Lizard | Average | 60.25% | 3974.93 |
|  | Median | 61.10% | 1378.00 |
| Frog | Average | 60.66% | 2161.58 |
|  | Median | 61.70% | 818.00 |

Supplementary Table S7. Differential alternative splicing events (DASE) among tissues at the same stage

| GO term ID | Tissues and time pairs | GO terms |
| --- | --- | --- |
| GO:0007422 | Adult_Heart_LegMuscle | peripheral nervous system development |
| GO:0010460 | Adult_Heart_LegMuscle | positive regulation of heart rate |
| GO:0045116 | Adult_Heart_LegMuscle | protein neddylation |
| GO:0045823 | Adult_Heart_LegMuscle | positive regulation of heart contraction |
| GO:0048168 | Adult_Heart_LegMuscle | regulation of neuronal synaptic plasticity |
| GO:0048172 | Adult_Heart_LegMuscle | regulation of short-term neuronal synaptic plasticity |
| GO:0002763 | Adult_Heart_Spleen | positive regulation of myeloid leukocyte differentiation |
| GO:0009913 | Adult_Heart_Spleen | epidermal cell differentiation |
| GO:0030852 | Adult_Heart_Spleen | regulation of granulocyte differentiation |
| GO:0045655 | Adult_Heart_Spleen | regulation of monocyte differentiation |
| GO:0045656 | Adult_Heart_Spleen | negative regulation of monocyte differentiation |
| GO:1902107 | Adult_Heart_Spleen | positive regulation of leukocyte differentiation |
| GO:1903708 | Adult_Heart_Spleen | positive regulation of hemopoiesis |
| GO:0002763 | Fetus_LegMuscle_Liver, Fetus_LegMuscle_Spleen | positive regulation of myeloid leukocyte differentiation |
| GO:0006591 | Fetus_LegMuscle_Liver, Fetus_LegMuscle_Spleen | ornithine metabolic process |
| GO:0009913 | Fetus_LegMuscle_Liver, Fetus_LegMuscle_Spleen | epidermal cell differentiation |
| GO:0010657 | Fetus_LegMuscle_Liver, Fetus_LegMuscle_Spleen | muscle cell apoptotic process |
| GO:0010660 | Fetus_LegMuscle_Liver, Fetus_LegMuscle_Spleen | regulation of muscle cell apoptotic process |
| GO:1902107 | Fetus_LegMuscle_Liver, Fetus_LegMuscle_Spleen | positive regulation of leukocyte differentiation |
| GO:1903708 | Fetus_LegMuscle_Liver, Fetus_LegMuscle_Spleen | positive regulation of hemopoiesis |
| GO:2000515 | Fetus_LegMuscle_Liver, Fetus_LegMuscle_Spleen | negative regulation of CD4-positive, alpha-beta T cell activation |
| GO:0002763 | M2_Heart_Spleen, M2_Kidney_LegMuscle | positive regulation of myeloid leukocyte differentiation |
| GO:0006591 | M2_Heart_Spleen, M2_Kidney_LegMuscle | ornithine metabolic process |
| GO:0009913 | M2_Heart_Spleen, M2_Kidney_LegMuscle | epidermal cell differentiation |
| GO:0010657 | M2_Heart_Spleen, M2_Kidney_LegMuscle | muscle cell apoptotic process |
| GO:0010660 | M2_Heart_Spleen, M2_Kidney_LegMuscle | regulation of muscle cell apoptotic process |
| GO:0030852 | M2_Heart_Spleen, M2_Kidney_LegMuscle | regulation of granulocyte differentiation |
| GO:0045655 | M2_Heart_Spleen, M2_Kidney_LegMuscle | regulation of monocyte differentiation |
| GO:0045656 | M2_Heart_Spleen, M2_Kidney_LegMuscle | negative regulation of monocyte differentiation |
| GO:1902107 | M2_Heart_Spleen, M2_Kidney_LegMuscle | positive regulation of leukocyte differentiation |
| GO:1903708 | M2_Heart_Spleen, M2_Kidney_LegMuscle | positive regulation of hemopoiesis |
| GO:0030852 | Y1_Kidney_LegMuscle | regulation of granulocyte differentiation |
| GO:0002763 | Y1_LegMuscle_Spleen, Y1_Kidney_LegMuscle | positive regulation of myeloid leukocyte differentiation |
| GO:0006591 | Y1_LegMuscle_Spleen, Y1_Kidney_LegMuscle | ornithine metabolic process |
| GO:0009913 | Y1_LegMuscle_Spleen, Y1_Kidney_LegMuscle | epidermal cell differentiation |
| GO:0010657 | Y1_LegMuscle_Spleen, Y1_Kidney_LegMuscle | muscle cell apoptotic process |
| GO:0010660 | Y1_LegMuscle_Spleen, Y1_Kidney_LegMuscle | regulation of muscle cell apoptotic process |
| GO:0045655 | Y1_LegMuscle_Spleen, Y1_Kidney_LegMuscle | regulation of monocyte differentiation |
| GO:0045656 | Y1_LegMuscle_Spleen, Y1_Kidney_LegMuscle | negative regulation of monocyte differentiation |
| GO:1902107 | Y1_LegMuscle_Spleen, Y1_Kidney_LegMuscle | positive regulation of leukocyte differentiation |
| GO:1903708 | Y1_LegMuscle_Spleen, Y1_Kidney_LegMuscle | positive regulation of hemopoiesis |
| GO:2000515 | Y1_LegMuscle_Spleen, Y1_Kidney_LegMuscle | negative regulation of CD4-positive, alpha-beta T cell activation |

Supplementary Table S8. DASE among developmental stages for the same tissue

| GO terms ID | Tissues and time pairs | GO terms |
| --- | --- | --- |
| GO:0003014 | Spleen_Fetus_Adult | renal system process |
| GO:0006977 | Spleen_Fetus_Adult | DNA damage response, signal transduction by p53 class mediator resulting in cell cycle arrest |
| GO:0009056 | Spleen_Fetus_Adult | catabolic process |
| GO:0016192 | Spleen_Fetus_Adult | vesicle-mediated transport |
| GO:0031571 | Spleen_Fetus_Adult | mitotic G1 DNA damage checkpoint |
| GO:0032355 | Spleen_Fetus_Adult | response to estradiol |
| GO:0044248 | Spleen_Fetus_Adult | cellular catabolic process |
| GO:0051049 | Spleen_Fetus_Adult | regulation of transport |
| GO:0060627 | Spleen_Fetus_Adult | regulation of vesicle-mediated transport |
| GO:0071392 | Spleen_Fetus_Adult | cellular response to estradiol stimulus |
| GO:0098801 | Spleen_Fetus_Adult | regulation of renal system process |
| GO:0072431 | Spleen_Fetus_Adut | signal transduction involved in mitotic G1 DNA damage checkpoint |
| GO:0002573 | Spleen_Fetus_M2 | myeloid leukocyte differentiation |
| GO:0006986 | Spleen_Fetus_M2 | response to unfolded protein |
| GO:0035966 | Spleen_Fetus_M2 | response to topologically incorrect protein |
| GO:0045667 | Spleen_Fetus_M2 | regulation of osteoblast differentiation |
| GO:0045669 | Spleen_Fetus_M2 | positive regulation of osteoblast differentiation |
| GO:0045778 | Spleen_Fetus_M2 | positive regulation of ossification |
| GO:0045842 | Spleen_Fetus_M2 | positive regulation of mitotic metaphase/anaphase transition |
| GO:0048660 | Spleen_Fetus_M2 | regulation of smooth muscle cell proliferation |
| GO:0048662 | Spleen_Fetus_M2 | negative regulation of smooth muscle cell proliferation |
| GO:1901970 | Spleen_Fetus_M2 | positive regulation of mitotic sister chromatid separation |
| GO:1902101 | Spleen_Fetus_M2 | positive regulation of metaphase/anaphase transition of cell cycle |
| GO:1902105 | Spleen_Fetus_M2 | regulation of leukocyte differentiation |
| GO:1902107 | Spleen_Fetus_M2 | positive regulation of leukocyte differentiation |
| GO:1903706 | Spleen_Fetus_M2 | regulation of hemopoiesis |
| GO:1903708 | Spleen_Fetus_M2 | positive regulation of hemopoiesis |
| GO:0006875 | Spleen_Fetus_Y1 | cellular metal ion homeostasis |
| GO:0010639 | Spleen_Fetus_Y1 | negative regulation of organelle organization |
| GO:0022605 | Spleen_Fetus_Y1 | oogenesis stage |
| GO:0031109 | Spleen_Fetus_Y1 | microtubule polymerization or depolymerization |
| GO:0097435 | Spleen_Fetus_Y1 | supramolecular fiber organization |
| GO:1902115 | Spleen_Fetus_Y1 | regulation of organelle assembly |
| GO:1902116 | Spleen_Fetus_Y1 | negative regulation of organelle assembly |
| GO:0030851 | Spleen_Fetus_M2 | granulocyte differentiation |
| GO:0030852 | Spleen_Fetus_M2 | regulation of granulocyte differentiation |
| GO:0002763 | Spleen_Fetus_M2 | positive regulation of myeloid leukocyte differentiation |
| GO:0002761 | Spleen_Fetus_M2 | regulation of myeloid leukocyte differentiation |
| GO:0045639 | Spleen_Fetus_M2 | positive regulation of myeloid cell differentiation |
| GO:0006914 | Spleen_Fetus_M2, Spleen_Fetus_Adult | autophagy |
| GO:0022617 | Spleen_Fetus_M2, Spleen_Fetus_Adult, Spleen_Fetus_Y1 | extracellular matrix disassembly |
| GO:0010715 | Spleen_Fetus_M2, Spleen_Fetus_Adult, Spleen_Fetus_Y1 | regulation of extracellular matrix disassembly |
| GO:1903053 | Spleen_Fetus_M2, Spleen_Fetus_Adult, Spleen_Fetus_Y1 | regulation of extracellular matrix organization |
| GO:0006782 | Liver_Fetus_M2 | protoporphyrinogen IX biosynthetic process |
| GO:0006783 | Liver_Fetus_M2 | heme biosynthetic process |
| GO:0009166 | Liver_Fetus_M2 | nucleotide catabolic process |
| GO:0014066 | Liver_Fetus_M2 | regulation of phosphatidylinositol 3-kinase signaling |
| GO:0014068 | Liver_Fetus_M2 | positive regulation of phosphatidylinositol 3-kinase signaling |
| GO:0016570 | Liver_Fetus_M2 | histone modification |
| GO:0030850 | Liver_Fetus_M2 | prostate gland development |
| GO:0046501 | Liver_Fetus_M2 | protoporphyrinogen IX metabolic process |
| GO:0046916 | Liver_Fetus_M2 | cellular transition metal ion homeostasis |
| GO:0048813 | Liver_Fetus_M2 | dendrite morphogenesis |
| GO:0055076 | Liver_Fetus_M2 | transition metal ion homeostasis |
| GO:0060996 | Liver_Fetus_M2 | dendritic spine development |
| GO:0097061 | Liver_Fetus_M2 | dendritic spine organization |
| GO:1901292 | Liver_Fetus_M2 | nucleoside phosphate catabolic process |
| GO:0007281 | Liver_Fetus_Y1 | germ cell development |
| GO:0048477 | Liver_Fetus_Y1 | oogenesis |
| GO:0051445 | Liver_Fetus_Y1 | regulation of meiotic cell cycle |
| GO:0051447 | Liver_Fetus_Y1 | negative regulation of meiotic cell cycle |
| GO:2000242 | Liver_Fetus_Y1 | negative regulation of reproductive process |
| GO:0009968 | Liver_Fetus_Y1, Liver_Fetus_Adult | negative regulation of signal transduction |
| GO:0010469 | Liver_Fetus_Y1, Liver_Fetus_Adult | regulation of receptor activity |
| GO:0016572 | Liver_Fetus_Y1, Liver_Fetus_Adult | histone phosphorylation |
| GO:0032269 | Liver_Fetus_Y1, Liver_Fetus_Adult | negative regulation of cellular protein metabolic process |
| GO:0043066 | Liver_Fetus_Y1, Liver_Fetus_Adult | negative regulation of apoptotic process |
| GO:0043069 | Liver_Fetus_Y1, Liver_Fetus_Adult | negative regulation of programmed cell death |
| GO:0047496 | Liver_Fetus_Y1, Liver_Fetus_Adult | vesicle transport along microtubule |
| GO:0051248 | Liver_Fetus_Y1, Liver_Fetus_Adult | negative regulation of protein metabolic process |
| GO:0072384 | Liver_Fetus_Y1, Liver_Fetus_Adult | organelle transport along microtubule |
| GO:0099518 | Liver_Fetus_Y1, Liver_Fetus_Adult | vesicle cytoskeletal trafficking |
| GO:1902532 | Liver_Fetus_Y1, Liver_Fetus_Adult | negative regulation of intracellular signal transduction |
| GO:0050965 | LegMuscle_Fetus_Y1, LegMuscle_Fetus_M2, LegMuscle_Fetus_Adult | detection of temperature stimulus involved in sensory perception of pain |
| GO:0006914 | LegMuscle_Fetus_Adult | autophagy |
| GO:0022617 | LegMuscle_Fetus_Y1 | extracellular matrix disassembly |
| GO:0010715 | LegMuscle_Fetus_Y1 | regulation of extracellular matrix disassembly |
| GO:1903053 | LegMuscle_Fetus_Y1 | regulation of extracellular matrix organization |
| GO:0010715 | Kidney_Fetus_Adult | regulation of extracellular matrix disassembly |
| GO:0022617 | Kidney_Fetus_Adult | extracellular matrix disassembly |
| GO:1903053 | Kidney_Fetus_Adult | regulation of extracellular matrix organization |
| GO:0002573 | Kidney_Fetus_M2 | myeloid leukocyte differentiation |
| GO:0002761 | Kidney_Fetus_M2 | regulation of myeloid leukocyte differentiation |
| GO:0002763 | Kidney_Fetus_M2 | positive regulation of myeloid leukocyte differentiation |
| GO:0030851 | Kidney_Fetus_M2 | granulocyte differentiation |
| GO:0030852 | Kidney_Fetus_M2 | regulation of granulocyte differentiation |
| GO:0045639 | Kidney_Fetus_M2 | positive regulation of myeloid cell differentiation |
| GO:1902105 | Kidney_Fetus_M2 | regulation of leukocyte differentiation |
| GO:1902107 | Kidney_Fetus_M2 | positive regulation of leukocyte differentiation |
| GO:1903708 | Kidney_Fetus_M2 | positive regulation of hemopoiesis |
| GO:0006914 | Kidney_Fetus_Y1 | autophagy |
| GO:0009166 | Heart_Fetus_M2, Heart_Fetus_Adult, Heart_Fetus_Y1 | nucleotide catabolic process |
| GO:1901292 | Heart_Fetus_M2, Heart_Fetus_Adult, Heart_Fetus_Y1 | nucleoside phosphate catabolic process |
| GO:0014066 | Heart_Fetus_M2 | regulation of phosphatidylinositol 3-kinase signaling |
| GO:0014068 | Heart_Fetus_M2 | positive regulation of phosphatidylinositol 3-kinase signaling |

Supplementary Table S9. The detailed information of 5 genes likely functioned in leg muscle development

| AS Events | AS type | NCBI_Gene_id | Paper_Gene_ID | Gene name | Description | Expression levels | | | | | Specific expressed tissue |
| --- | --- | --- | --- | --- | --- | --- | --- | --- | --- | --- | --- |
|  |  |  |  |  |  | Heart | Kidney | Leg Muscle | Liver | Spleen |  |
| ASE015400 | RI | gene1208 | XTS00001233 | BBS5 | Bardet-Biedl syndrome 5 | 309.67 | 0.25 | 3648.42 | 0.08 | 29.17 | Leg Muscle |
| ASE015399 | RI | gene1208 | XTS00001233 |  |  | 345.92 | 0.25 | 3730.75 | 0.17 | 30.17 | Leg Muscle |
| ASE023031 | SE | gene1208 | XTS00001233 |  |  | 11.75 | 0.00 | 0.58 | 0.00 | 0.00 | Heart |
| ASE023031 | SE | gene1208 | XTS00001233 |  |  | 2.17 | 5.25 | 1.75 | 1.75 | 1.67 | None |
| ASE015399 | RI | gene1208 | XTS00001233 |  |  | 0.67 | 2.08 | 2.25 | 1.50 | 3.08 | None |
| ASE015400 | RI | gene1208 | XTS00001233 |  |  | 0.33 | 1.75 | 1.75 | 1.58 | 3.17 | None |
| ASE022118 | SE | gene19405 | XTS00019408 | NRG4 | Neuregulin 4 | 1.00 | 0.08 | 2.17 | 0.00 | 0.17 | Leg Muscle |
| ASE022117 | SE | gene19405 | XTS00019408 |  |  | 0.00 | 0.00 | 0.08 | 0.00 | 0.00 | None |
| ASE022116 | SE | gene19405 | XTS00019408 |  |  | 6.17 | 8.50 | 8.08 | 0.50 | 0.75 | None |
| ASE022114 | SE | gene19405 | XTS00019408 |  |  | 9.50 | 12.67 | 13.58 | 1.00 | 0.92 | None |
| ASE022116 | SE | gene19405 | XTS00019408 |  |  | 1.00 | 1.58 | 1.75 | 0.17 | 0.08 | None |
| ASE022114 | SE | gene19405 | XTS00019408 |  |  | 1.42 | 2.42 | 2.25 | 0.17 | 0.00 | None |
| ASE022118 | SE | gene19405 | XTS00019408 |  |  | 2.58 | 2.08 | 4.58 | 0.42 | 0.58 | None |
| ASE011959 | RI | gene20480 | XTS00020483 | IP6K3 | inositol hexakisphosphate kinase 3 | 26.75 | 0.42 | 243.75 | 2.08 | 6.92 | Leg Muscle |
| ASE011959 | RI | gene20480 | XTS00020483 |  |  | 0.33 | 0.00 | 9.08 | 0.08 | 0.00 | Leg Muscle |
| ASE019954 | SE | gene20480 | XTS00020483 |  |  | 1.75 | 0.17 | 16.50 | 0.25 | 0.58 | Leg Muscle |
| ASE019954 | SE | gene20480 | XTS00020483 |  |  | 3.00 | 0.08 | 31.25 | 0.17 | 0.33 | Leg Muscle |
| ASE017471 | SE | gene2657 | XTS00002682 | AMPD1 | Adenosine Monophosphate Deaminase 1 | 0.00 | 0.00 | 1834.00 | 0.00 | 2.00 | Leg Muscle |
| ASE017471 | SE | gene2657 | XTS00002682 |  |  | 0.75 | 0.00 | 294.50 | 0.00 | 0.25 | Leg Muscle |
| ASE009306 | RI | gene2657 | XTS00002682 |  |  | 0.00 | 0.17 | 257.92 | 0.00 | 0.08 | Leg Muscle |
| ASE009306 | RI | gene2657 | XTS00002682 |  |  | 0.00 | 0.00 | 0.58 | 0.00 | 0.00 | None |
| ASE021119 | SE | gene10670 | XTS00010684 | DYSF | dysferlin | 4.08 | 0.00 | 14.67 | 0.00 | 0.08 | Leg Muscle |
| ASE002068 | A3SS | gene10670 | XTS00010684 |  |  | 10.17 | 0.00 | 39.08 | 0.00 | 1.08 | Leg Muscle |
| ASE017023 | SE | gene10670 | XTS00010684 |  |  | 10.17 | 0.00 | 39.08 | 0.00 | 1.08 | Leg Muscle |
| ASE013379 | RI | gene10670 | XTS00010684 |  |  | 15.33 | 18.42 | 30.42 | 8.75 | 20.17 | None |
| ASE017023 | SE | gene10670 | XTS00010684 |  |  | 25.08 | 31.08 | 49.08 | 13.50 | 37.00 | None |
| ASE002068 | A3SS | gene10670 | XTS00010684 |  |  | 18.67 | 15.83 | 39.33 | 6.50 | 24.08 | None |
| ASE016598 | SE | gene10670 | XTS00010684 |  |  | 29.33 | 34.83 | 34.42 | 17.75 | 19.58 | None |
| ASE017065 | SE | gene10670 | XTS00010684 |  |  | 3.67 | 3.58 | 4.75 | 2.67 | 3.92 | None |
| ASE021119 | SE | gene10670 | XTS00010684 |  |  | 11.00 | 1.75 | 17.08 | 2.67 | 9.92 | None |
| ASE017065 | SE | gene10670 | XTS00010684 |  |  | 29.58 | 13.50 | 59.42 | 7.42 | 14.92 | None |
| ASE013379 | RI | gene10670 | XTS00010684 |  |  | 9.42 | 0.92 | 23.67 | 1.08 | 6.42 | None |
| ASE016598 | SE | gene10670 | XTS00010684 |  |  | 44.50 | 2.25 | 81.83 | 6.42 | 38.00 | None |


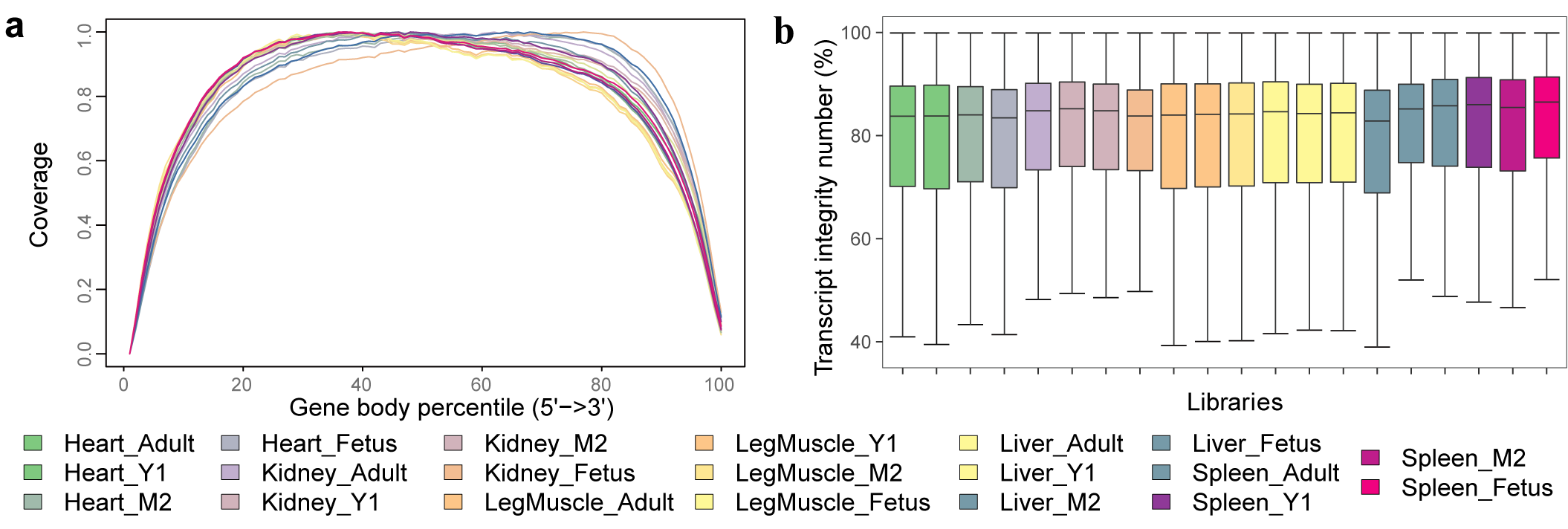


Supplementary Figure S1. **Reliability evaluation of RNA-seq data for identifying and analyzing the goat AS landscape**. (**a**) The distribution of mapped reads along goat annotated genes. (**b**) The percentage of goat annotated transcripts covered by at least four uniquely mapped reads.


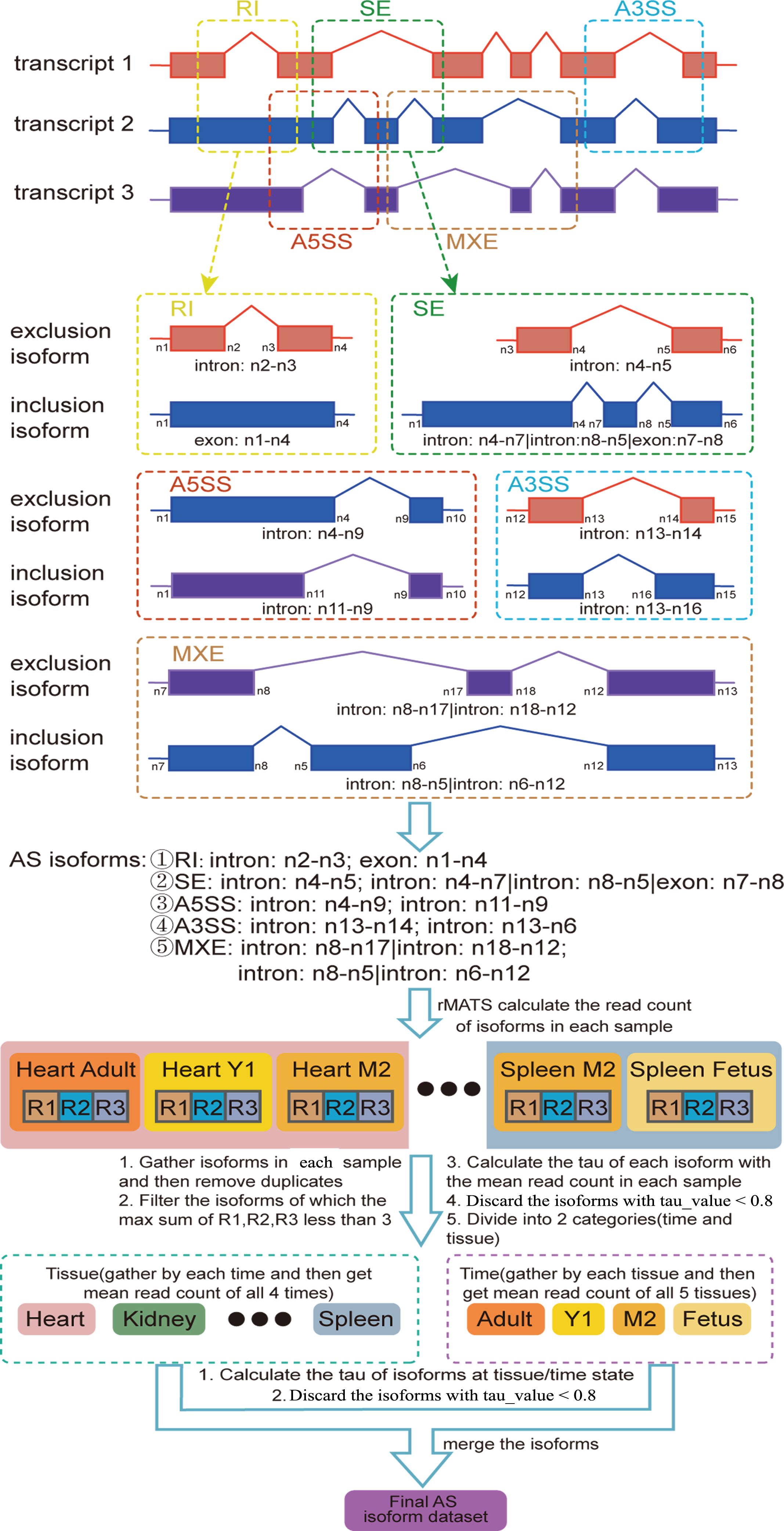


Supplementary Figure S2. The pipeline for identification of tissue- or developmental stage- specific AS events


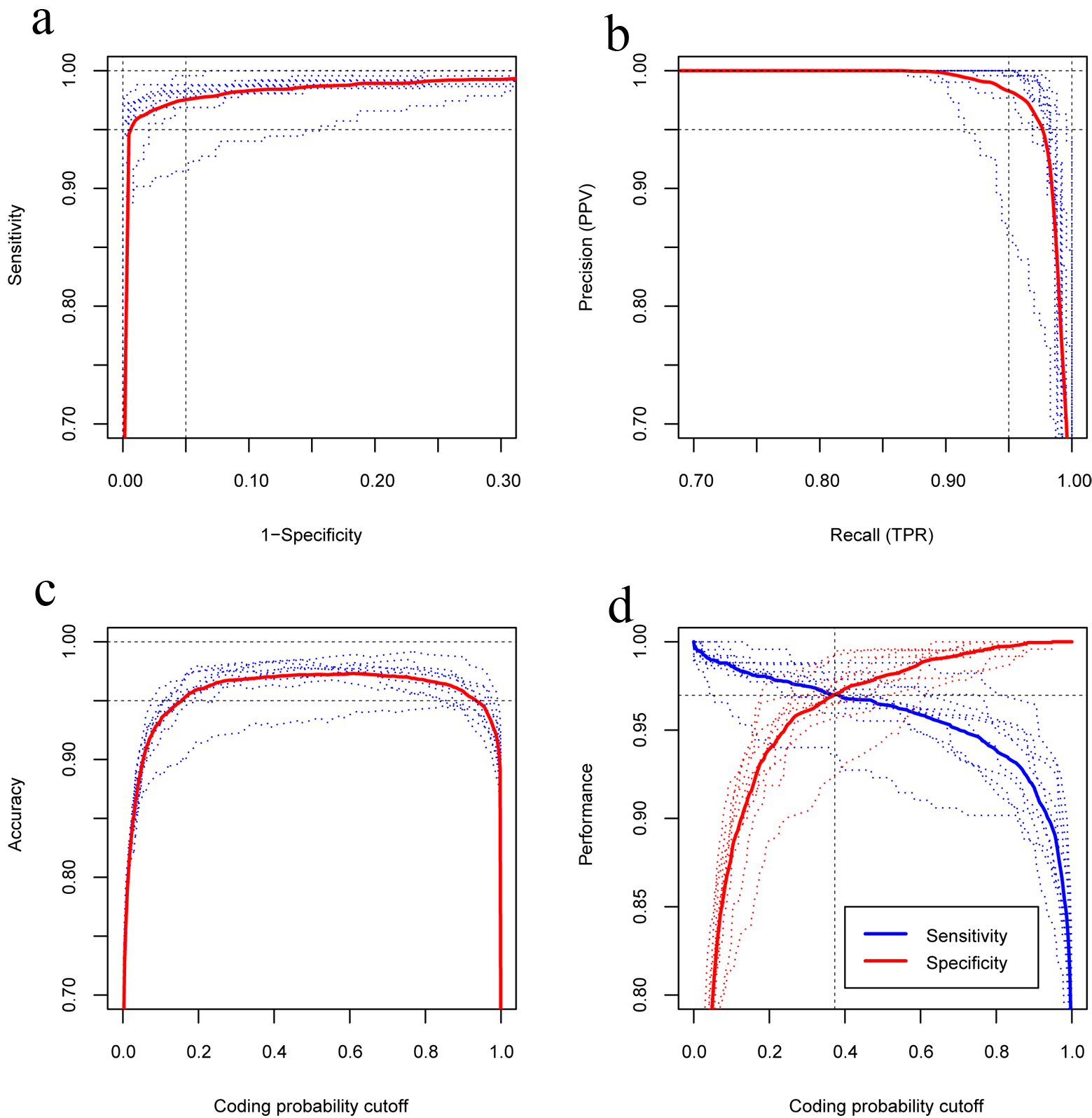


Supplementary Figure S3. The sensitivity and specificity in discrimination of lncRNAs and protein-coding genes using CPAT with a 0.40 cutoff. (A) ROC curve. (B) PR (precision-recall) curve. (C) Accuracy vs cutoff value. (D) Two graphic ROC curve to determine the optimum cutoff value.
